# Global Transcriptome and Coexpression Network Analyses Reveal New Insights Into Somatic Embryogenesis in Hybrid Sweetgum (*Liquidambar styraciflua* × *Liquidambar formosana*)

**DOI:** 10.3389/fpls.2021.751866

**Published:** 2021-11-22

**Authors:** Shuaizheng Qi, Ruirui Zhao, Jichen Yan, Yingming Fan, Chao Huang, Hongxuan Li, Siyuan Chen, Ting Zhang, Lisheng Kong, Jian Zhao, Jinfeng Zhang

**Affiliations:** ^1^College of Biological Science and Biotechnology, Beijing Forestry University, Beijing, China; ^2^Department of Biology, Centre for Forest Biology, University of Victoria, Victoria, BC, Canada

**Keywords:** hybrid sweetgum, somatic embryogenesis, transcriptome, coexpression network, qRT-PCR

## Abstract

Somatic embryogenesis (SE) is a process of somatic cells that dedifferentiate to totipotent embryonic stem cells and generate embryos *in vitro*. Despite recent scientific headway in deciphering the difficulties of somatic embryogenesis, the overall picture of key genes, pathways, and co-expression networks regulating SE is still fragmented. Therefore, deciphering the molecular basis of somatic embryogenesis of hybrid sweetgum remains pertinent. In the present study, we analyzed the transcriptome profiles and gene expression regulation changes via RNA sequencing from three distinct developmental stages of hybrid sweetgum: non-embryogenic callus (NEC), embryogenic callus (EC), and redifferentiation. Comparative transcriptome analysis showed that 19,957 genes were differentially expressed in ten pairwise comparisons of SE. Among these, plant hormone signaling-related genes, especially the auxin and cytokinin signaling components, were significantly enriched in NEC and EC early. The K-means method was used to identify multiple transcription factors, including *HB-WOX, B3-ARF, AP2/ERF*, and *GRFs* (growth regulating factors). These transcription factors showed distinct stage- or tissue-specific expression patterns mirroring each of the 12 superclusters to which they belonged. For example, the *WOX* transcription factor family was expressed only at NEC and EC stages, *ARF* transcription factor was expressed in EC early, and *GRFs* was expressed in late SE. It was noteworthy that the *AP2/ERF* transcription factor family was expressed during the whole SE process, but almost not in roots, stems and leaves. A weighted gene co-expression network analysis (WGCNA) was used in conjunction with the gene expression profiles to recognize the genes and modules that may associate with specific tissues and stages. We constructed co-expression networks and revealed 22 gene modules. Four of these modules with properties relating to embryonic potential, early somatic embryogenesis, and somatic embryo development, as well as some hub genes, were identified for further functional studied. Through a combination analysis of WGCNA and K-means, SE-related genes including *AUX22, ABI3, ARF3, ARF5, AIL1, AIL5, AGL15, WOX11, WOX9, IAA29, BBM1, MYB36, LEA6, SMR4* and others were obtained, indicating that these genes play an important role in the processes underlying the progression from EC to somatic embryos (SEs) morphogenesis. The transcriptome information provided here will form the foundation for future research on genetic transformation and epigenetic control of plant embryogenesis at a molecular level. In follow-up studies, these data could be used to construct a regulatory network for SE; Key genes obtained from coexpression network analysis at each critical stage of somatic embryo can be considered as potential candidate genes to verify these networks.

## Introduction

Chinese sweetgum (*Liquidambar formosana* Hance) is distributed in most temperate and subtropical regions of China and is used mainly for medicinal and ornamental purposes (Sun et al., 2016). American sweetgum (*Liquidambar styraciflua* L.) is a common southern American hardwood that has become an important feedstock for the timber and paper industries. These two species are interfertile even after 10 million years of separation (Vendrame et al., 2001). Some clones of hybrid sweetgum (*L. styraciflua* × *L. formosana*) have biomass productivity that is superior that of either parent species due to faster growth rates and higher wood density, demonstrating obvious heterosis (Merkle, 2018). Thus, the breeding of hybrid sweetgum is expected to have great potential value for the wood industry. Furthermore, potentially scalable SE-based propagation systems have been reported for both *L. styraciflua* (Merkle et al., 1998) and hybrid sweetgum (*L. styraciflua* × *L. formosana*) (Vendrame et al., 2001; Merkle et al., 2010). We successfully developed a SE system for hybrid sweetgum from hybrid immature seed explants. This system will be the foundation for breeding research and large-scale propagation of hybrid sweetgum. However, the mechanism of its SE remains unclear, which strongly hampers the advancement of breeding research and efficient propagation.

Somatic embryogenesis is one of the biotechnological tools which makes somatic embryos (SEs), similar in morphology to zygotic embryo without fertilization. Somatic embryos is bipolar structure which has both shoot apex and root apex (Dodeman et al., 1997; Ikeda et al., 2006). Also, SE is a multi-step regeneration process that includes three stages: embryonic induction, embryonic development, and somatic embryo development (Elhiti et al., 2013). First, a individual somatic cell or group of somatic cells under suitable *in vitro* conditions generates embryogenic or non-embryogenic cells. Some of these non-embryogenic callus (NEC) cells can differentiate further into embryogenic callus (EC) cells (Wen et al., 2020). The formation of callus is usually considered as a manifestation of the dedifferentiated cellular state. Second, the callus/proembryogenic cell mass (PEM) undergoes a series of biochemical and morphological changes to form SEs through redifferentiation or eventually to form a whole plant (Jiménez, 2005; Quiroz-Figueroa et al., 2006). The stages of somatic embryos include globular embryos (GE), heart-shaped embryos (HE), torpedo-shaped embryos (TE), and cotyledonary embryos (CE), all of which closely resemble those of zygotic embryos, both morphologically and temporally (Dodeman et al., 1997); hence, molecular information generated by studying the SE pathway can be used to explain the dynamic molecular interactions that occur during SE (Zimmerman, 1993; Quiroz-Figueroa et al., 2006). Somatic embryos has been reported in many plant species, including woody plants (more than 180 species of angiosperms and more than 35 species of gymnosperms) such as *Theobroma cacao* (Alemanno et al., 1996) and *Hevea brasiliensis* (Michaux-Ferrière and Carron, 1989), and has become an attractive regeneration method for *in vitro* mass propagation, germplasm preservation, and genetic improvement (Karami and Saidi, 2010).

Many studies have examined the biochemical and physiological changes of SE in varieties of plant species with the goal of understanding the mechanisms and functions of regulation of gene expression related to SE. These studies have identified genes that are differentially expressed in SE, highlighted pathways that may be involved in SE, and revealed molecular or protein markers for SE (Mantiri et al., 2008; Chen et al., 2020). Many studies have reported that some genes and proteins play a key role in SE. The identification of key genes in model plants could help us understand the mechanism underlying SE. The expression of several key regulators, including *Somatic Embryogenesis Receptor-Like Kinase 1 (SERK1)* gene, which was overexpression increases the efficiency of somatic cell initiation in Arabidopsis (Hecht et al., 2001). *LEAFY COTYLEDON2* (*LEC2*) (Gaj et al., 2005; Rupps et al., 2016), *ABSCISIC ACID INSENSITIVE3* (*ABI3*), *FUSCA3* (*FUS3*), which encode B3 domain transcription factors that are key regulators of embryogenesis (Freitas et al., 2019), were identified and verified as direct target genes of *AGAMOUS-LIKE15* (*AGL15*). Genes identified as targets of the B3 genes are also targets of *AGL15* (Zheng et al., 2009). The homologous domain transcription factor *WUS*, which can regulate the formation and maintenance of stem cells, was an early marker gene for *SAM* initiation in embryos (Weigel and Jürgens, 2002; Elhiti et al., 2010). *BABY BOOM* (*BBM*) encodes an *APETALA2* (*AP2*) domain transcription factor that is preferentially expressed in developing embryos and seeds. Overexpression of *BBM* can trigger spontaneous formation of somatic embryo (Maulidiya et al., 2020). In addition to these genes, *WUSCHEL homeobox 2* (*WOX2*) (Tvorogova et al., 2015), *AUXIN RESPONSE FACTOR 19* (*ARF19*) (Xiao et al., 2020), and LATE EMBRYOGENESIS ABUNDANT (LEA) protein (Wickramasuriya and Dunwell, 2015; Jamaluddin et al., 2017) also play key roles in SE. However, the gene changes and regulations in the SE of hybrid sweetgum remain unknown.

In the present research, we analyzed the transcriptome profiles and gene regulation changes in the SE process of hybrid sweetgum using a combination of K-means and WGCNA methods. Some genes were identified as being related to the NEC, EC, and redifferentiation of SE. These included genes related to hormones, embryogenesis, and various transcription factors (TFs). This is the first report of SE transcriptomes in hybrid sweetgum, and the results improve our understandings of the molecular mechanisms in the SE of hybrid sweetgum. Some new molecular marker genes were identified, which enriched the molecular regulatory network during SE and provided a new perspective for improving the efficiency of SE through genetic transformation. Furthermore, the results of this research will be provide a valuable resource for future studies and will be helpful to the research in this field, especially hybrid sweetgum breeding programs.

## Materials and Methods

### Plant Material and RNA Extraction

**FIGURE 1:**
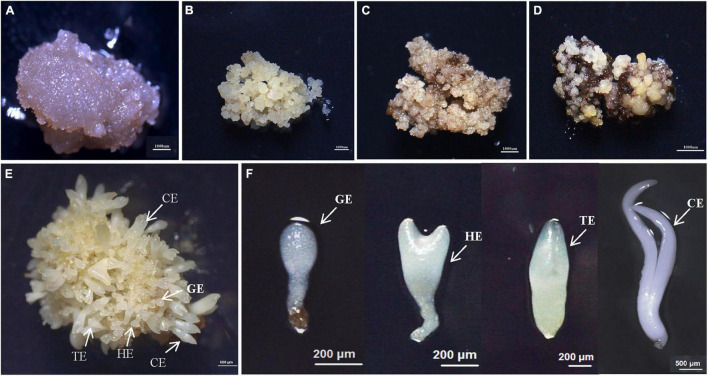
The synchronized cultures during hybrid sweetgum SE. **(A)** NEC: non-embryogenic callus; **(B)** EC: friable-embryogenic callus; **(C)** PEM1: 20 days of pro-embryogenic mass; **(D)** PEM2: 45 days of pro-embryogenic mass; **(E)** embryogenic callus with somatic embryos; **(F)** Somatic embryos at different developmental stages (globular embryo, heart-shaped embryo, torpedo-shaped embryo, cotyledonal embryo). A–D Bars = 1000 μm; E Bars = 800 μm; F Bars = 200 μm, 500 μm.

**FIGURE 2:**
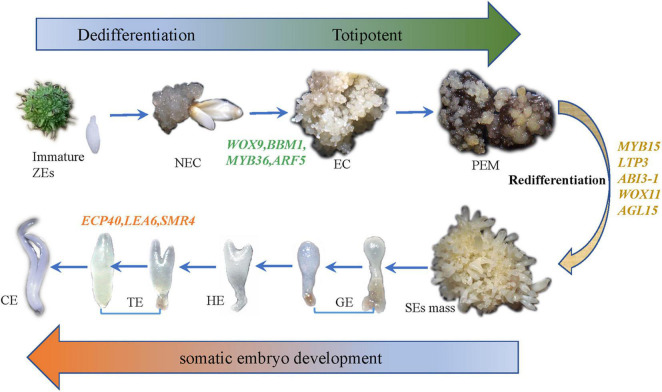
The whole experimental diagram and hypothesized molecular mechanisms of SE.

To obtain hybrid seeds, we performed controlled pollinations between *L. formosana* and *L. styraciflua* trees. Developing fruits (multiples of capsules) were collected from the *L. styraciflua* trees. Whole fruits were surface-disinfested using the following sequence: rinse with water for 30 min, soaked in 75% ethanol for 1min and sodium hypochlorite for 4 min on the ultra-clean table, and cleaned with sterile distilled water for 5–6 times. The fruits were then dissected aseptically with a scalpel to remove the immature seeds. Each seed was nicked with a scalpel and cultured, ten seeds per plate, on induction medium: A modified Blaydes medium (Merkle et al., 1998) with 1.0 mg/L 2,4-dichlorophenoxyacetic acid (2,4-D), 0.5 mg/L 6-benzylaminopurine (6-BA), and 1.0 g/L casein hydrolysate (CH). The medium was Explants were transferred to fresh media of the same composition monthly until NEC (callus that were completely NEC or had both NEC and EC) started to form. Callus were transferred to fresh medium every 3 weeks. EC were transferred to embryo development medium –modified Blaydes basal medium without plant growth regulators (PGRs) – and incubated in the dark for 3–4 weeks, until embryos began to emerge from the PEMs. Individual somatic embryos were selected and cultured (16 per 90-mm plastic petri plate) on fresh medium of the same composition, also in the dark. When the embryos had enlarged to 2–3 mm in length, they were transferred to medium lacking PGRs and CH and incubated under cool white fluorescent light (140 μmol m^–2^ s^–1^) for germination. The cultures at different developmental stages, including NEC, friable EC, PEM after maturation cultured for 20 days (PEM1), PEM after maturation cultured for 45 days (PEM2), GE, HE, TE, CE, roots (R), stems (S), and leaves (L) were sampled, as shown schematically in Figures 1, 2. We collected the NEC, EC, PEM1, PEM2, GE, HE, TE, CE, and vegetative organs of plantlet samples (roots, stems, and leaves) from tissues in each of the three replicates and stored these at –80°C for RNA extraction.

### Illumina Sequencing and Data Analysis

A total of 1 μg of RNA per sample was used as the input material for the RNA sample preparations. Three experimental replicates per sample were used for RNA library construction. RNA-Seq libraries were generated using the NEBNext Ultra RNA Library Prep Kit for Illumina (New England Biolabs, United States) following the manufacturer’s recommendations, and index codes were added to attribute sequences to each sample. After sequencing, the raw data were filtered to remove adaptor contamination and low-quality reads. All clean reads were then mapped to the hybrid sweetgum genome using the HISAT2 program. The mapped reads of each gene were extracted using HISAT2 (Kim et al., 2015). We filtered the significantly differentially expressed genes (DEGs) using | log_2_ (fold change)| ≥ 1.5 and *P*-value < 0.01 between seven pairwise comparisons: NEC_vs_EC, EC_vs_PEM1, PEM1_vs_PEM2, PEM2_vs_GE, GE_vs_HE, HE_vs_TE, TE_vs_CE, CE_vs_R, CE_vs_L, and CE_vs_S. Gene expression levels were normalized using the [fragments per kilobase of transcript per million fragments mapped (FPKM)] (Florea et al., 2013) method. All samples had more genes expressed at the level of 10 to 100 FPKM, and only 1196 to 1426 genes were identified at levels higher than 100 FPKM (Supplementary Table 1).

### K-Means Clustering Analysis

K-means clustering with Euclidean distance in MeV 4.9 software yielded 50 clusters based on inputs of log_2_ relative FPKM values. In total, 13,574 genes residing in clusters with even more obvious tissue- or stage-specific expression trends were selected to make the final 35 K-means clusters. Only 18 of the 35 clusters are shown in Figure 5. TFs were extracted from the respective clusters. The log_2_ relative FPKM value of each TF was used to produce heat maps in TBtools (Chen et al., 2018) (Figure 5B).

### Co-expression Network Construction and Hub Gene Search

For co-expression network analysis, the WGCNA (Zhang and Horvath, 2005; Langfelder and Horvath, 2008) package was used. Based on the log_2_ (1 + FPKM) values, a matrix of pairwise (spearman correlation coefficient) SCCs between all pairs of genes was generated and transformed into an adjacency matrix (a matrix of connection strengths) using the following formula: connection strength (adjacency value) = |(1 + correlation)/2|^β^. Here, β represents the soft threshold for the correlation matrix, which gives greater weight to the strongest correlations while maintaining gene–gene connectivity. Based on the scale-free topology criterion, the β value was selected as 13 (Supplementary Figure 1A). The adjacent matrix was transformed into topological overlap (TO) matrix by TOM similarity algorithm, and the hierarchical clustering of genes was carried out based on TO similarity. The dynamic tree-cutting algorithm was used to cut the hierarchal clustering dendrogram, decomposed/merged branches and defined modules to achieve a stable number of clusters (Supplementary Figure 1B) (Langfelder and Horvath, 2008). For each module, a summary profile (module eigengene) was calculated via principal component analysis. Further, the modules with higher TO values (average TO for all genes in a given module) than those of modules comprised of randomly selected genes were retained. Finally, the gene interactions in key modules were visualized using Cytoscape software, and a gene co-expression network based on the hit number was constructed. Cytoscape software (Shannon et al., 2003) was used to visualize the gene interactions of key modules and constructed the gene co-expression network based on hit number. Considering the expression information, we marked these genes and confirmed the top 20 hub unigenes per group. The Cytoscape working data are available in Supplementary Table 2.

### Gene Ontology and Pathway Enrichment Analysis

Gene Ontology (GO) enrichment analysis for DEG sets was performed using Cytoscape (BiNGO plug-in) (Maere et al., 2005). The P-value for enrichment was calculated for each represented GO term and corrected using the Benjamini–Hochberg error correction method. The GO terms exhibiting a corrected (after adjusting with the false discovery rate) P-value ≤ 0.05 were considered to be significantly enriched. Furthermore, pathway enrichment analysis of different sets of genes was performed using MapMan (v 3.6.0RC1) categories (significance value ≤ 0.05). MapMan bins of supercluster genes were assigned using the Mercator pipeline for automated sequence annotation (Thimm et al., 2004)^1^.

### Quantitative Real-Time PCR Analysis

Quantitative real-time polymerase chain reaction (qRT-PCR) was conducted on 33 RNA samples that were used in the preparation of sequencing libraries using a CFX Connect^®^ Real-Time PCR Detection System (CFX Connect, Bio-Rad, Munich, Germany). Each sample included three biological replicates. Nine genes that specifically highly expressed in non-embryogenic callus, embryogenic callus and somatic embryo development stage, respectively, were selected for qRT-PCR to verify the RNA-seq results, and then reverse transcribed of 1 μg total RNA into cDNA using a *TransScript* One-Step DNA Removal and cDNA Synthesis SuperMix Kit (Beijing Transgen Biotech Co. Ltd., Beijing, China). Each PCR reaction mixture contained 10 μl of Trans Start Top Green qPCR SuperMix (Beijing Transgen Biotech Co. Ltd., Beijing, China), 1 μl of template cDNA, 0.4 μl of forwarding primer, 0.4 μl of reverse primer (Supplementary Table 3), and 8.2 μl ddH_2_O to a final volume of 20 μl with three technical replicates of each gene. The amplification condition for qRT-PCR was under the following program: denaturation at 94°C for 30 s, 40 cycles at 94°C for 5 s, 60°C for 15 s, 72°C for 15 s, with a melt cycle from 65 to 95°C. The apple EF1-α gene (Zheng et al., 2017) served as the internal standard. The relative gene expression in each sample was normalized to EF1 Ct value and calculated using the 2^–ΔΔ^ ^Ct^ method.

## Results

### Morphological Characterization of Somatic Callus

To study the SE of hybrid sweetgum, immature seeds of hybrid sweetgum (*L. styraciflua* × *L. formosana*) were tissue culture-induced to NEC. The NEC cells could be differentiated from the EC cells by visual discrimination. The NECs were dense and watery, white to brown in color, and had disorganized growth (Figure 1A). The ECs were friable, light yellow in color, and had organized globular structures that gave rise to SEs (Figure 1B). Once embryogenic cells have formed, they continue to proliferate and form PEMs. Browning takes place in PEMs when they are cultured on maturation medium for 20 days (Figure 1C), and the browning callus can regrow a new globular structure callus that continues to culture for 25 days (Figure 1D). Finally, globular, heart, torpedo, and cotyledon stage somatic embryos appear after 60 days (Figures 1E,F, 2).

### Transcriptome Sequencing and Gene Expression Profiles

To provide a clear understanding of hybrid sweetgum at the transcription level, we sequenced the 33 cDNA libraries constructed from 11 developmental stages: NEC, EC, PEM1, PEM2, GE, HE, TE, CE, roots (R), stems (S), and leaves (L) (Figure 1). In total, 998,501,629 clean reads (containing about 299.46 G of nucleotides) were obtained with quality checking. After alignment with the hybrid sweetgum reference genome, the clean data were mapped to the reference genome with a mapping ratio varying from 48,451,523 (76.36%) to 38,189,210 (81.49%). Among these, the unique mapping ratio varied from 47,112,326 (74.25%) to 37,112,232 (79.19%). In each sample, more than 91.08% had a base score of Q30 or higher. In our transcriptome, alternative splicing (AS) events were predicted by ASprofile. In total, 1,528,934 AS events, including alternative 5’ splicing, alternative 3’ splicing, exon skipping, intron retention, and the other 12 types of AS, were checked across the 33 samples. HE2 has the largest number of AS events (51,015), and the smallest was detected in L2 (37,151). A summary of the mapping statistics obtained for each sample is given in Supplementary Table 4.

**FIGURE 3:**
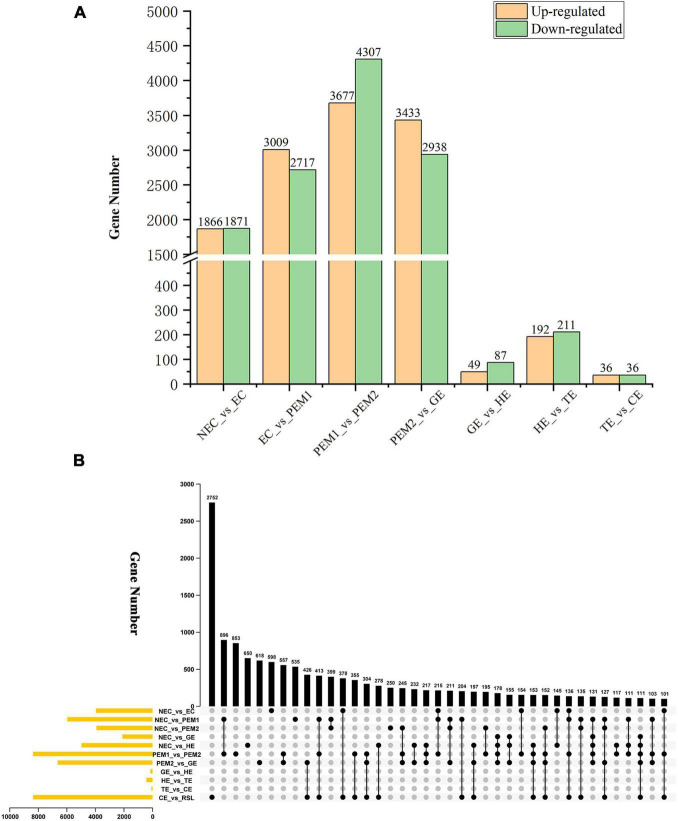
Statistical analysis of differentially expressed unigenes in NEC, PEMs and different SE stages. **(A)** Statistic of Up/Down regulated genes in pairwise comparisons of NEC_vs_EC, EC_vs_PEM1, PEM1_vs_PEM2, PEM2_vs_GE, GE_vs_HE, HE_vs_TE and TE_vs_CE. **(B)** The Upset plot of expressed genes in eleven pairwise comparisons.

The aim of this study was to reveal the potential key genetic factors related in the SE of hybrid sweetgum. Among these seven comparisons (Figure 3A), 3737, 5726, 7984, 6371, 136, 403, and 72 DEGs were identified, respectively. In comparison with NEC, EC had 1866 upregulated and 1871 downregulated genes. In comparison with EC, PEM1 had 3009 upregulated and 2717 downregulated genes. In comparison with PEM1, PEM2 had 3367 upregulated and 4307 downregulated genes. In comparison with PEM1, GE had 3433 upregulated and 2938 downregulated genes. In comparison with GE, HE had 49 upregulated and 87 downregulated genes. In comparison with HE, TE had 192 upregulated and 211 downregulated genes. In comparison with TE, CE had 36 upregulated and 36 downregulated genes. The Upset plot was also created to find the overlapped genes in the comparisons of eleven pairwise comparisons of hybrid sweetgum SE (Figure 3B). As shown in Figure 3B, 853 genes were exclusive to PEM1_vs_PEM2. 618 genes were exclusive to PEM2_vs_GE. Moreover, 598 genes were found in NEC_vs_EC. DEG analysis showed that the hybrid sweetgum transcriptome changed significant dynamically during SE, especially during the transition period from PEM1 to PEM2.

### Functional Classification of Differentially Expressed Genes Based on Gene Ontology and Kyoto Encyclopedia of Genes and Genomes Analyses

To evaluate the potential functions of the DEGs, we used GO term assignment to classify the functions of the DEGs in pairwise comparisons under three main GO categories: biological process, cellular component, and molecular function (Supplementary Figure 2). In all pairwise comparisons, the term with the largest proportion in biological process was metabolic process, followed by cellular process, single-organism process, biological regulation, respond to stimulus and localization, the term with the largest proportion in cellular component were cell and cell part, followed by membrane and organelle, the term with the largest proportion in molecular function was catalytic activity, followed by binding, transporter activity, structural molecular activity and nucleic acid binding transcription factor activity. In all pairwise comparisons among the functional groups, “metabolic process,” “cell,” “catalytic activity” terms were dominant. To investigate the biological pathways of the DEGs, we used the Kyoto Encyclopedia of Genes and Genomes (KEGG) database to classify the functions of the DEGs with emphasis on biological pathways (Supplementary Figure 3). According to the KEGG annotations, 701 DEGs (NEC_vs_EC) were assigned to 120 pathways, 1308 DEGs (EC_vs_PEM1) were assigned to 125 pathways, 1403 DEGs (PEM1_vs_PEM2) were assigned to 123 pathways, 1389 DEGs (PEM2_vs_GE) were annotated to 123 pathways, 18 DEGs (GE_vs_HE) were annotated to 21 pathways, 100 DEGs (HE_vs_TE) were assigned to 63 pathways, and 17 DEGs (TE_vs_CE) were annotated to 18 pathways. The pathway enrichment analysis showed that the annotated changes in NEC_vs_EC were mainly related to plant hormone signal transduction and fatty acid elongation. Among them, the genes annotated to fatty acid metabolism were up-regulated. The annotated changes in EC_vs_PEM1 were primarily involved in ribosomes and oxidative phosphorylation. The annotated changes in PEM1_vs_PEM2 were primarily involved in glutathione metabolism and phenylpropanoid biosynthesis. The annotated changes in PEM2_vs_GE were primarily involved in ribosomes and fatty acid elongation. Among them, the genes annotated to ribosome biogenesis in eukaryotes were up-regulated. The annotated changes in GE_vs_HE were mostly involved in brassinosteroid biosynthesis and starch and sucrose metabolism. Among them, the genes annotated to brassinosteroid biosynthesis pathway were significantly up-regulated, while the genes with alpha-linolenic acid metabolism were significantly down-regulated. The annotated changes in HE_vs_TE were primarily involved in phenylpropanoid biosynthesis and biosynthesis of amino acids. Among them, the genes annotated to phenylalanine metabolism were down-regulated. The annotated changes in TE_vs_CE were primarily involved in linoleic acid metabolism and alpha-linoleic acid metabolism. Among them, the genes annotated to alpha-linolenic acid metabolism and linolenic acid metabolism were down-regulated.

### Differential Expression Analysis of Plant Hormone Signaling Pathway-Related Genes During Hybrid Sweetgum Somatic Embryos

Based on the KEGG and GO annotations, plant hormone signal transduction, zeatin biosynthesis, and IAA biosynthesis were the representative pathways in our study. A large number of genes involved in auxin (95 DEGs) and cytokinin (40 DEGs) biosynthesis and signal transduction pathways were differentially expressed when comparing EC with NEC and different SE stages.

**FIGURE 4:**
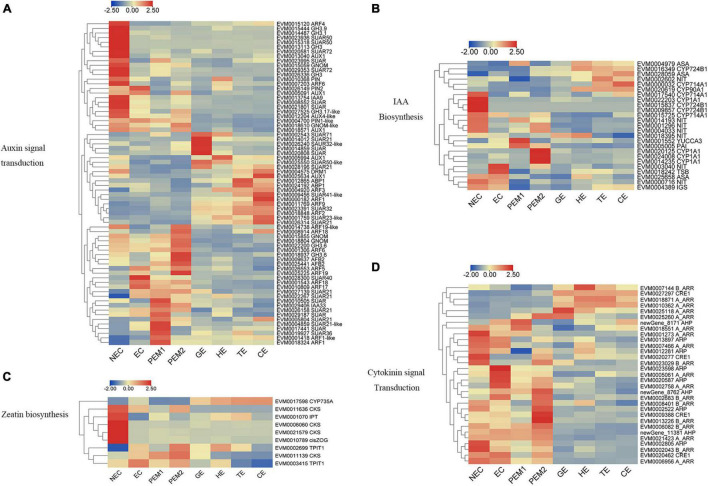
Heatmap of the differentially expressed genes in auxin and cytokinin signaling pathway during hybrid sweetgum SE. **(A)** Auxin signal transduction; **(B)** IAA biosynthesis; **(C)** Zeatin biosynthesis; **(D)** Cytokinin signal transduction. The heatmap was clustered by pearson method of TBtools software. Heatmap indicate the gene expression level by Log2[FPKM] with a color scale, each row represents a single gene, the IDs and names of selected DEGs are indicated to the right of the histograms, and each column represents a sample.

For example, the expression levels of *PIN1-like, PIN2, AUX1, ARFs* (*ARF5, ARF17, ARF18, ARF19*), *GH3* (*GH3.6, GH3.1, GH3.17*), and five *SAUR* genes—all involved in auxin signal transduction—were significantly upregulated from NEC to EC. Nevertheless, *AUX1, GNOM, GNOM-like, DRM1, PIN, PIN2, AUX4-like, TIR1, IAA9, ARFs* (*ARF4, ARF6, ARF19-like*), *GH3* (*GH3.9, GH3.1, GH3, GH3.17-like*), and *SAUR* (*SAUR50, SAUR72*) were mainly expressed in the NEC stage and downregulated in EC. From the EC to PEM and GE stages, *GNOM, AFB2, GH3.6, IAA33, ARFs* (*ARF1, ARF1-like, ARF6, ARF18, ARF19, ARF19-like*), and two *SAUR32-like* genes showed noteworthy upregulated expression (Figure 4A). In indole-3-acetic acid (IAA) biosynthesis, *NIT* and *TSB* were upregulated in EC. *CYP714A1, CYP90A1, CYP1A1, CYP724B1*, and three *NIT* genes showed an NEC-specific expression pattern. One *YUCCA3*, three *CYP1A1*, and one *PAI* gene were upregulated in PEM, and *CYP724B1, CYP714A1, CYP90A1, ASA*, and *NIT* remained upregulated during the development stage of SEs (Figure 4B).

**FIGURE 5:**
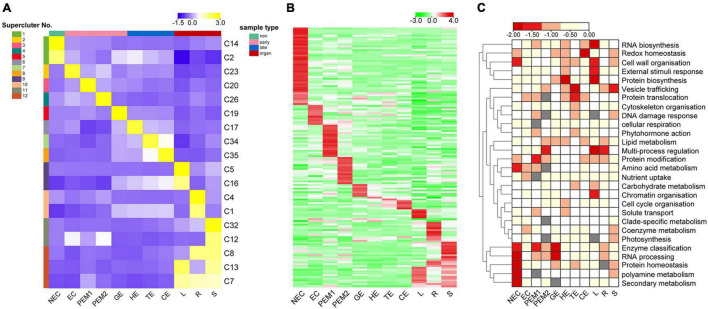
K-means clustering reveals unique tissue- and/or stage-specific expression trends. **(A)** Eighteen clusters representing 6,815 genes are shown with distinct stage- and tissue-specific expression patterns. Averaged Normalized FPKM of all the genes in each cluster was used to generate the heat map. These clusters are further grouped into 12 superclusters shown to the right. Heat map indicate the gene expression level by gradient color scale in which yellow indicates high expression. **(B)** Clustering based on Normalized FPKM of 142 transcription factors. Red scale indicates high expression. **(C)** Enriched MapMan bins shown to the right for each of the 12 superclusters. Significant enrichment is indicated by low P values.

As shown in Figure 4C, Three *CKS*, one *IPT*, and one *Cis-ZOG* family genes involved in *cis*-zeatin O-glycosylation were highly expressed in NEC and significantly downregulated from NEC to EC and stages of somatic embryo development. Two *TPIT1* and one *CKS* gene were notably downregulated from NEC to EC and PEM and interestingly decreased during somatic embryogenesis, and the expression trends of these genes were consistent in studies on somatic embryogenesis of Longan (Chen et al., 2020). One *CYP735A was* upregulated in somatic embryo development. For the cytokinin signaling pathway, four *A_ARR*, two *AHP*, three *B_ARR*, and *CRE1* genes were mainly expressed in NEC but downregulated in EC. Two *A_ARR*, one *B_ARR*, and three *AHP* genes were upregulated in EC. One *A_ARR*, four *B_ARR*, and three *AHP* genes were upregulated in PEM, and one *CRE1*, one *B_ARR*, and three *A_ARR* genes showed upregulated expression during the development stage of SEs (Figure 4D). Analysis of differential gene expression patterns indicated that genes related to cytokinin signal transduction and synthesis were differentially expressed during somatic embryogenesis, which may be involved in the regulation of somatic embryogenesis in hybrid sweetgum. Multiple sequences of the same hormone-related genes were compared, and the highest alignment rate was 97.21%, while the lowest was 17.58%. Most of the gene sequences were different, indicating that these genes were different isoforms (Supplementary Figure 4).

### Identification of Spatiotemporal Expression Trends Across Hybrid Sweetgum Transcriptomes

Thirty-five K-means clusters (containing 19,957 genes) with the most obvious tissue- and stage-specific expression trends were selected. Among these, 18 clusters representing 6815 genes are shown in Figure 5A and Supplementary Table 5. The 18 clusters can be divided into four large clusters, which are NEC, early SE (EC, PEM1, PEM2, GE), somatic embryo development (GE, HE, TE, CE) and vegetative organ (L, R, S). Early SE-specific gene clusters (C23, C20, C26, and C19) and embryo development-specific gene clusters (C17, C19, C34, and C35) were identified (Figure 5A). The NEC and vegetative organ categories had highly unique transcriptomes; almost 60% of the genes with tissue-specific expression were found in the NEC (C2 and C14) and vegetative organ (C1, C4, C5, C7, C8, C12, C13, C16, and C32) groups. However, a unique transcriptome was also found during the somatic embryo development stage; almost 10% of the genes with stage-specific expression were in embryo development.

Clusters with similar expression trends were combined into 12 superclusters (Figure 5A), and specific genes or pathway enriched in specific superclusters were identified by MapMan Bins (Figure 5C and Supplementary Table 5). The top abundant MapMan bin for the NEC-specific supercluster (supercluster 1) was polyamine metabolism, protein homeostasis, and secondary metabolism. As shown in Figure 5C, the biological functions associated with early somatic embryogenesis, nutrient uptake, enzyme classification, multi-process regulation, protein modification, and RNA processing were enriched in EC, PEM, and GE. Genes that participated in major vesicle trafficking, redox homeostasis, and regulation of proteins, including biosynthesis, homeostasis, and translocation, were particularly enriched in the somatic embryo development stage.

Among the 6815 genes that make up the aforementioned 12 superclusters (Figure 5A), 142 are TFs belonging to 60 families (Figure 5B and Supplementary Table 5). These TFs exhibit different stage- or tissue-specific expression patterns, and the dynamic and stage- or tissue-specific expression patterns of these TFs may reflect the pivotal roles that they play. For instance, the EC-specific (supercluster 2) cluster has four *RLK/Pelle* family kinases, one *C2C2* gene, and one ethylene response factor gene with potential roles in embryogenic competence. Interestingly, the embryo development-specific (superclusters 5–8) cluster has one *GRF* gene, indicating that this gene plays a key role in the morphogenesis of SEs.

### Co-expression Network Analysis With Weighted Gene Co-expression Network Analysis

**FIGURE 6:**
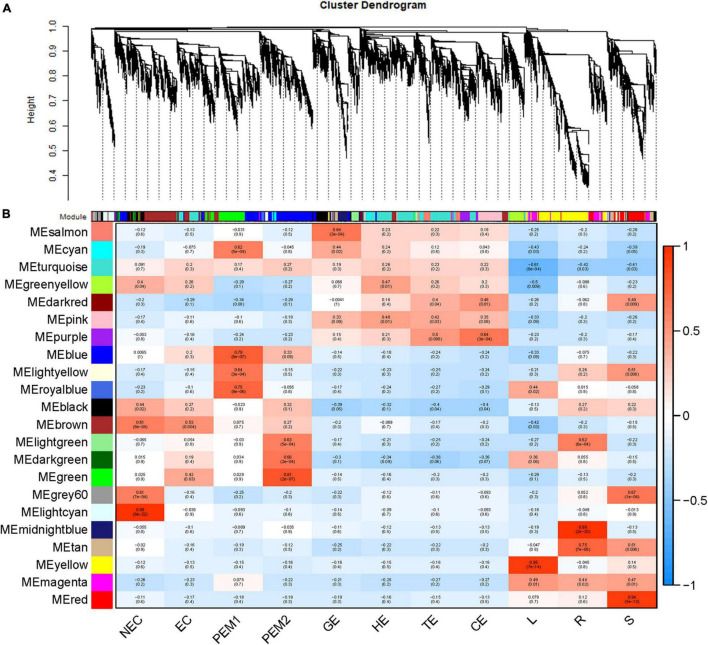
WGCNA of genes in eleven tissues. **(A)** Hierarchical cluster tree showing coexpression modules identified by WGCNA. Each leaf in the tree is one gene. The major tree branches constitute 22 modules labeled by different colors. **(B)** Module-tissue association. Each row corresponds to a module. The number of genes in each module is indicated on the left. Each column corresponds to a specific tissue. The color of each cell at the row-column intersection indicates the correlation coefficient between the module and the tissue type. A high degree of correlation between a specific module and the tissue type is indicated by red.

As an alternative analysis tool, weighted gene coexpression network analysis (WGCNA), was adopted. WGCNA is a systems biology approach that aims to understand entire gene networks rather than single genes. In this study, we constructed a co-expression network based on the pairable correlation of gene co-expression trends in all sample tissues. Modules were defined as clusters of genes that were highly interrelated; The correlation coefficient between genes in the same cluster was high. This analysis resulted in 22 different modules, color-coded in the dendrogram (Figure 6A and Supplementary Figure 1B), where each branch constitutes a module and each branch is a gene (Figure 6A and Supplementary Table 2). The first principal component of a given module was the module eigengene, which represented the gene expression profile of the module. Due to the tissue-specific expression profile of the eigengene, 22 characteristic genes of 22 different modules were associated with different tissue types. In comparison with other co-expression modules, for instance, the module with the highest correlation with NEC was the lightcyan module (*p* = 8 × 10^–22^, *r* = 0.99), whereas genes in the green module had a higher correlation with EC (*p* = 3 × 10^–2^, *r* = 0.42) than NEC, except for PEM2. Genes in the blue module were comparatively highly correlated with early SE (EC, PEM1, PEM2). Interestingly, three co-expression modules, the pink, salmon, and purple modules, showed relatively high correlation (*r* ≥ 0.15) with somatic embryo development stages (GE, HE, TE, CE) (Figure 6B). Many of the modules were correlated with more than one vegetative organ of the plantlet; however, a few were correlated with only a specific vegetative organ of the plantlet. For example, the yellow module was specifically correlated (*p* = 7 × 10^–14^, *r* = 0.95) with leaf tissue, whereas the midnight-blue and red modules were specifically correlated with root (*p* = 2 × 10^–20^, *r* = 0.98) and stem (*p* = 5 × 10^–13^, *r* = 0.94) tissues, respectively (Figure 6B).

**FIGURE 7:**
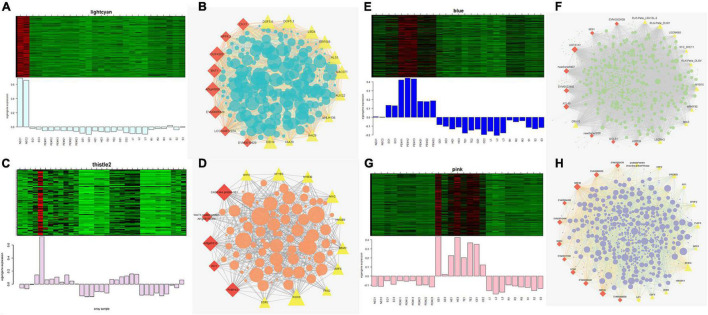
Expression profile and transcriptional regulatory network associated with the tissue-specific modules. Heatmaps of **(A)** lightcyan module (NEC), **(C)** thistle modules (EC), **(E)** blue module (early SE), **(G)** pink module (somatic embryo development stages) show the expression profile of all the coexpressed genes in the modules (labeled on top). The color scale represents Z-score. Bar graphs (below the heat maps) show the consensus expression pattern of the coexpressed genes in each module. **(B)** Correlation network of genes in lightcyan module (NEC). **(D)** Correlation network of genes in thistle module (EC). **(F)** Correlation network of genes in blue module (early SE). **(H)** Correlation network of genes in pink module (somatic embryo development stages). Red diamonds indicate hub genes, and yellow triangles indicate transcription factors.

Figures 7A,C,E,G show the eigengene expressions for the lightcyan (NEC), thistle (EC) modules, blue (EC, PEM1, PEM2) and pink (GE, HE, TE, CE) modules. The heatmap shows the expression profiles of all co-expressed genes from the blue, pink, lightcyan, and thistle modules. WGCNA can also be used to drawn gene networks, where each node represents a gene and the edges are the connecting lines between nodes, represent co-expression-related genes. The node gene with the highest connectivity, as the hub gene, may play a key role in different modules. The lightcyan, thistle, blue and pink module networks are shown in Figures 7B,D,F,H. Some of the more interconnected node genes are those of TFs. Only the top 20 genes with high connectivity were selected, and 10 TFs representing different families were obtained, including the *WRKY, RLK*-*Pelle, MYB*, and *STE*_*STE11* TFs. Another nine hub genes were also identified as key proteins or enzymes that play a key role in early SE (blue module) (Figure 7F and Supplementary Table 2). In the pink module network, 77 of the 965 genes encode TFs. Selecting only the top 23 genes showing high connectivity weight resulted in 11 TFs representing distinct families, including the *AP2/ERF-ERF, C2C2, C3H, bHLH, GRF*, and *bZIP* TFs. Another 12 hub genes, namely c*yclin-dependent protein kinase inhibitor* (*SMR4*), *seed biotin-containing protein* (*SBP65*), and *late embryogenesis abundant protein 6* (*LEA6*), are involved in regulation of the cell cycle, seed germination capacity, and embryo development ending in seed dormancy (Figure 7H and Supplementary Table 2). In the lightcyan module network (Figure 7B), 37 of the 416 genes encode TFs. Selecting only the top 19 genes showing high connectivity weight indicated 11 TFs representing distinct families, including the *AUX/IAA* (EVM0009101; EVM0009101), *C2C2-Dof, GNAT, bHLH, AP2/ERF-ERF*, and *NAC* TFs (Supplementary Table 2). Another eight hub genes, namely *protein NRT1/PTR FAMILY 4.3* (*NPF4.3*), *cytokinin dehydrogenase 1* (*CKX1*), and *brassinosteroid-related acyltransferase 1* (*BAT1*), are involved in regulation of transmembrane transporter activity, cytokinin metabolic processes, and response to brassinosteroids. Interestingly, after we performed the co-expression analysis of the data again, we observed that one module displayed opposite expression patterns between the two types of calluses (NEC and EC) having different embryogenic potentials. For example, the thistle module showed low transcript abundance in NEC but high abundance in EC. In this module, 38 of the 212 genes encode TFs. Selecting only the top 15 genes showing high connectivity weight revealed 10 TFs representing distinct families, including the *HB-WOX, AP2/ERF-AP2, MYB, B3-ARF, AP2/ERF-ERF, FAR1*, and *C2H2* TFs. Another five hub genes, namely, *peptidyl-prolyl cis-trans isomerase* (*FKBP43*), *CASP-like protein 1E1, beta-fructofuranosidase, insoluble isoenzyme 1* (*INV1*), *GDSL esterase/lipase* (*At5g45910*), and *WAT1-related protein* (*At1g25270*) were involved in regulation of peptidyl-prolyl cis-trans isomerase activity, protein histidine kinase binding, carbohydrate storage, lipid catabolic processes, and transmembrane transporter activity (Figure 7D). Interestingly, the *FKBP43* gene is a fundamental protein that regulates cell division, adhesion and elongation throughout the plant development and embryogenesis and required for the spatial organization of apical meristems. These results suggest that the *FKBP43* gene may play a key role in the proliferation and maintenance of embryogenic ability of embryogenic callus.

Gene Ontology (GO) enrichment analysis was performed for each module using Cytoscape (BiNGO plug-in) (Maere et al., 2005) highlighted important biological processes represented by a range of co-expressed genes. The blue module formed a cluster of 2632 DEGs enriched in functions related to phosphorus metabolic processes (phosphate metabolic process, phosphorylation, and protein amino acid phosphorylation) and amine metabolic processes (aminoglycan catabolic process, chitin metabolic process, and aminoglycan catabolic process) (Supplementary Figure 5C). The pink module formed a cluster of 965 DEGs enriched in functions related to RNA metabolic processes (RNA processing, rRNA metabolic process, rRNA processing, and ncRNA processing) and responses to stress (response to cold, response to temperature stimulus, and response to abiotic stimuli) (Supplementary Figure 5D). The lightcyan module formed a cluster of 416 DEGs enriched in functions related to the catabolic process, carbohydrate catabolic process, polysaccharide catabolic process, pectin catabolic process, and cotyledon vascular tissue pattern formation (Supplementary Figure 5A). The thistle module obtained from the other WGCNA analysis formed a cluster of 211 DEGs enriched in functions related to regulation of metabolic processes (transcription, macromolecule metabolic process, RNA metabolic process, and nucleic acid metabolic process) and developmental processes (reproductive developmental process, anatomical structure development, and multicellular organismal development) (Supplementary Figure 5B).

### Quantification of the Level of Relative Expression

To verify the reliability of transcriptome data, twenty genes related to SE were selected.

**FIGURE 8:**
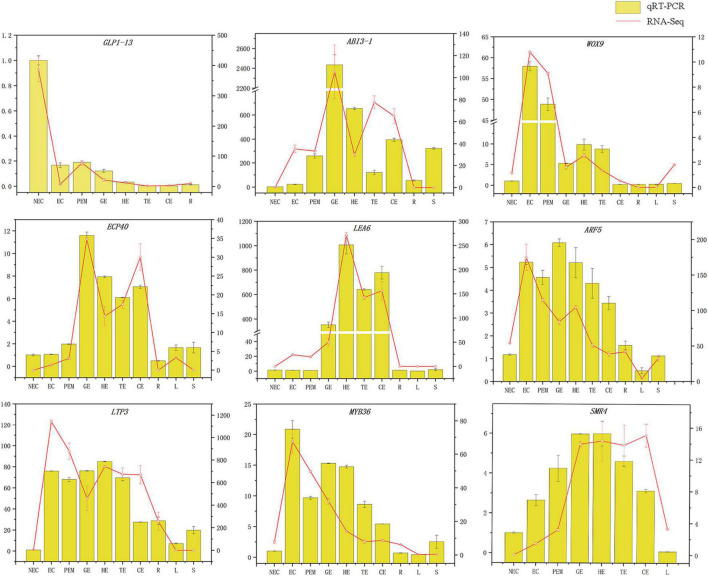
Comparison of expression profiles of ten representative genes as measured by RNA-seq and qRT-PCR. Columns represent expression determined by qRT-PCR (left y-axis), while lines represent expression by RNA-seq in FPKM values (right y-axis). The x-axis in each chart represents the ten stages (NEC-S). For qRT-PCR assay, the mean was calculated from three biological replicates each with three technical replicates (*n* = 9). For RNA-seq, each point is the mean of three biological replicates.

The *GLP1-13* (EVM0020840), *ARF5* (EVM0026553), *ABI3-1* (EVM0020968), *LTP3* (EVM0021586), *WOX9* (EVM0013624), *LEA6* (EVM0000925), *SMR4* (EVM0007351), *ECP40* (EVM0003818), and *MYB36* (EVM0015487) genes with high specific expression in various stages of hybrid sweetgum transcriptome were selected as marker genes to measure expression by qRT-PCR (Supplementary Table 3). The qRT-PCR analysis showed that expression levels during different SE phases changed between the genes (Figure 8). *WOX9* and *MYB36*, as selected molecular marker genes, were almost not expressed in NEC, while they mainly expressed during early SE, with the highest expression at EC stage and then decreased in the mature somatic embryos of SE. This showed that those molecular markers played a key role in the maintenance and acquisition of embryogenic ability. Meanwhile, *ECP40, ABI3-1*, and *LEA6* were highly expressed or up-regulated during somatic embryo development processes, and minimally or undetected in NEC. Which suggested that those marker genes may promote the hybrid sweetgum somatic embryo development. *LTP3, SMR4*, and *ARF5* were highly expressed or up-regulated during the whole SE processes, and low expression in NEC and vegetative organ (R, L, S), indicated that those molecular markers played a key role in SE. In addition, the transcription level of *GLP1-13* was highly and specific expressed in NEC, and this gene may inhibit the transition from non-embryonic to embryonic cells. qRT-PCR verification of SE-related genes also demonstrated a high correlation between RNA-seq and qRT-PCR data.

## Discussion

Because hybrid sweetgum (*L. styraciflua* × *L. formosana*) is a tree species that grows faster and has higher wood density than its parents, its SE is becoming increasingly important. We successfully developed an SE system for hybrid sweetgum from hybrid seed explants. However, the mechanism of its SE remained unknown. In this study, we performed transcriptomic analyses of 11 types of tissues and stages of SE. The RNA sequencing (RNA-seq) data were analyzed using two different bioinformatics methods, K-means and WGCNA. This analysis yielded several new insights, including the identification of some time-specific modules, hub genes, and transcription factors.

### Important Roles of Auxin and Cytokinin in Hybrid Sweetgum Somatic Embryos

As we all known that auxin and cytokinin are important factors in plant cell division, differentiation, and SE induction. In this study, auxin and cytokinin signal transduction and biosynthesis related genes were significantly expressed during somatic embryogenesis, among which there were many auxin signal transduction genes, such as *SUAR* (small auxin up RNA) family genes had the largest number. However, the levels of endogenous auxin and cytokinin are influenced by their exogenous application (Vondráková et al., 2011; Xu et al., 2013). Auxin is considered to be the central regulator of SE, which may be due to the establishment of an auxin gradient during SE induction (Yang et al., 2012). To date, the effect of exogenous auxin during SE has been reported in several studies (Yang et al., 2012; Chu et al., 2017). In accordance with these results, we found a mass of auxin-responsive genes differentially expressed between tissues with contrasting embryogenic potential and at particular stages in hybrid sweetgum SE.

In samples of EC, contents of many auxin related genes, such as *ARF17, ARF18* and *AUX1*, were significantly higher than those of NEC. The expression levels of *ARF9* and *ARF2* were significant during somatic embryo development, and increased gradually with somatic embryo development, and reached the highest in cotyledon embryo maturation. It is well-known that members of the *ARF* and *AUX/IAA* transcription regulator/signaling families act in concert to regulate the expression of responsive genes (Vanneste and Friml, 2009; Kieffer et al., 2010). The level of endogenous IAA has been correlated with PEM formation and high-frequency SE competency (Zhou et al., 2017). The balance of free IAA and IAA conjugates during SE induction of *Coffea canephora* was critical for embryogenic potential (Ayil-Gutierrez et al., 2013). The conjugation of auxin is synthesized by the *GH3* family (Chen et al., 2010), and *PIN1* plays a fundamental role in maintaining the embryonic auxin gradients (Feher, 2019). To summarize, we found that some of these family genes (*PIN1-like, PIN2, AUX1, ARFs, GH3*, and *SAUR*) had lower expression in NEC. Most of them were dramatically upregulated in EC and downregulated during SE, indicating that the auxin-responsive genes played an important role in hybrid sweetgum SE. Tryptophan (Trp)-dependent IAA biosynthesis is an important pathway in higher plants, and exogenous application of doses of Trp and IAA produced similar enhancements during rice SE (Siriwardana and Nabors, 1983). In our study, the expression levels of *TSB* genes, key genes in Trp synthesis, were drastically upregulated in EC and downregulated during SE. No Trp synthesis gene showed NEC specificity with high FPKM, suggesting that the level of Trp was higher during SE than during NEC.

Aside from auxin being a main inducer of SE, the use of exogenously supplied cytokinin to induce SE has been well established in many species (Sagare et al., 2000; Cabrera-Ponce et al., 2018). Large numbers of transcripts involved in zeatin biosynthesis and signal transduction are differentially expressed during cotton SE (Xu et al., 2013). Furthermore, endogenous cytokinin levels have been shown to be higher in SE than in NEC (Lai and Chen, 2002). In our study, from NEC to EC, a total of 31 DEGs were implicated in cytokinin signal transduction, including four *CRE1* (two upregulated and one downregulated), seven *B-ARR* (two upregulated and five downregulated), and 11 *A-ARR* (two upregulated and four downregulated) genes. In the zeatin synthesis pathway, *TRIT1* was upregulated from NEC to EC, and most of the *CisZOG, IPT*, and *CKS* were downregulated in EC and remained low during SE. However, IAA and zeatin biosynthesis- and signal transduction-related genes regulate SE in a complex and comprehensive way. Additional study of these genes in hybrid sweetgum SE is required.

### Key Role of Somatic Embryos-Related Genes During Hybrid Sweetgum Somatic Embryos

**FIGURE 9:**
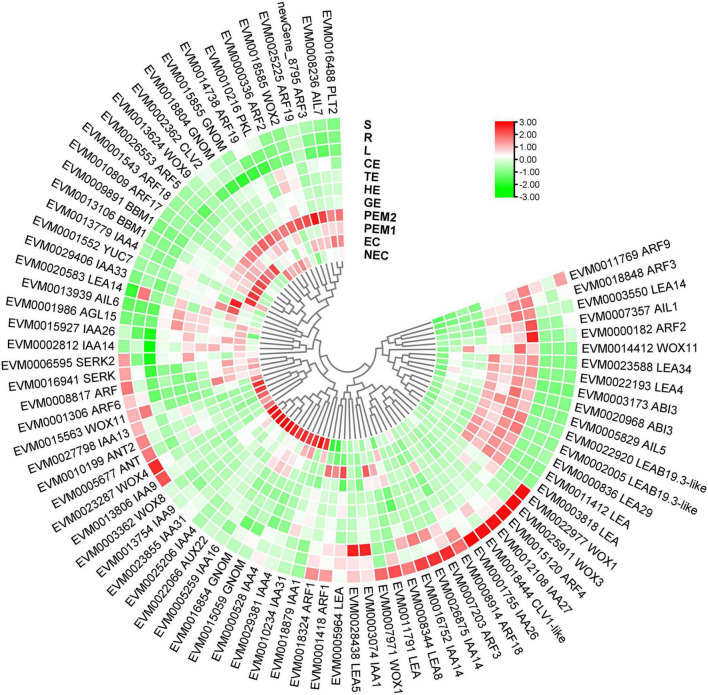
Heatmap of the differentially expressed SE-related genes during hybrid sweetgum SE. The heatmap was clustered by pearson method of TBtools software. Heatmap indicate the gene expression level by Log2[FPKM] with a color scale, each row represents a single gene, the IDs and names of selected DEGs are indicated to the outside of fan, and each circle represents a sample.

Many genes and TFs have been reported as key factors in SE induction. Some TFs, including *LEC1, LEC2, FUS3, ABI3, WOX9, WOX2*, and *BBM*, were identified as molecular marker genes for early microspore embryogenesis of *Brassica napus* (Malik et al., 2007). In our study, 11 molecular markers were TFs (*AUX22, ABI3, ARF3, ARF5, AIL1, AIL5, AGL15, WOX11, WOX9, IAA29, BBM1*, and *MYB36*), and some of these gene functions on embryogenesis have been well characterized in various plants (Figure 9 and Supplementary Table 6). *LEC2, ABI3*, and *FUS3* are B3 domain-containing transcription factors; *ABI3* is highly expressed in embryogenic masses of *Coffea arabica*, and it has been speculated that its activity is related to embryogenic potential (Freitas et al., 2019). The *AP2/ERF* family has two very important members, namely *BBM* and *PLT2*, which have been reported to play important roles in microspore, somatic, and zygotic embryogenesis (Tsuwamoto et al., 2007). Overexpression of *BBM* can trigger spontaneous formation of SEs in *Arabidopsis thaliana* and *B. napus*. Therefore, *BBM1* can serve as a marker gene for embryogenic callus cells in *B. napus* (Boutilier et al., 2002). In this assay, *BBM* and *ABI3* were highly expressed during the SE processes. However, *BBM1* was more highly expressed during the embryonic processes, and it can be used as a remarkable marker for maintenance of totipotent potential in hybrid sweetgum. To date, The *MADS-box* gene of *AGL15* is expressed preferentially during embryonic development, and the protein accumulation reaches the highest level; it is expressed at lower levels at the later stage of germination (Fernandez et al., 2000; Yang and Zhang, 2010). Notably, *AGL15* was shown to be beneficial to somatic embryo development and promoted the production of secondary embryonic callus from cultured zygotic embryos (Harding et al., 2003). The homologous domain transcription factor *WUS*, which can regulate the formation and maintenance of stem cells, was an early marker gene for *SAM* initiation in embryos (Weigel and Jürgens, 2002). *STIMPY/WOX9* played an key role in increasing the proliferation rate and preventing premature differentiation in emerging seedlings (Wu et al., 2005). *WOX2* and *WOX9* were highly expressed at the early stage of SE in *Picea abies*, and may play a role together in conifer embryo model (Palovaara et al., 2010). *WOX11* and *WOX12* genes promoted the division of procambium cells and played a key role in callus and adventitious root formation (Liu et al., 2014). In this assay, *WOX9* was specifically expressed in EC and downregulated during SE. *WOX11* was highly expressed throughout SE and almost not expressed in NEC or vegetative organs. Hence, these genes might play an important role in hybrid sweetgum SE.

### Identification of Weighted Gene Co-expression Network Analysis Module Genes Highly Associated With Hybrid Sweetgum Somatic Embryos

As an effective data mining method, WGCNA can screen genes related to specific traits and conduct modular classification on large samples to obtain co-expression modules with high biological significance (Zhao et al., 2010). Weighted gene co-expression network analysis has been used widely for plants in recent years (Zhao et al., 2020; Liu et al., 2021; Panahi and Hejazi, 2021). Therefore, we constructed a hybrid sweetgum gene co-expression network using a WGCNA approach and identified co-expression modules using transcriptome data. In total, according to the gene expression pattern, 22 modules were identified, several of which showed functional specificity at different stages (Figure 6). Then, to investigate the functional biological roles of these DEGs, BiNGO (Maere et al., 2005) software was used (Supplementary Table 2).

Our analysis identified many genes that were differentially expressed in the thistle module (EC). Of those with annotations, we found that the expression of many TFs, such as the *HB-WOX, AP2/ERF-AP2, MYB, B3-ARF, AP2/ERF-ERF, FAR1*, and *C2H2* TFs, increased in EC. TFs played an important role in regulating plant differentiation and development. For instance, *MYB* TFs may regulate cell proliferation and elongation (Wen et al., 2020). Several *R2R3-MYB* TFs are barely expressed in NEC, significantly upregulated from NEC to EC, and maintained at high levels during early SE in longan (Chen et al., 2020). *WUSCHEL-related homeobox* (*WOX*) genes encoding plant-specific homeobox TFs play key roles in plant development and growth (Li et al., 2019), can regulate stem cell maintenance and formation, and are an early molecular marker for shoot apical meristem (SAM) initiation in embryos (Weigel and Jürgens, 2002). *AP2/ERF* TFs function in the response to phytohormones and/or biotic or abiotic stresses. *BBM* and *ESR2*, which are known to be associated with cellular pluripotency in *Arabidopsis* (Matsuo et al., 2011; Kulinska-Lukaszek et al., 2012), are also involved in pluripotency acquisition in hybrid sweetgum callus. Therefore, these genes may play a key role in the acquisition and maintenance of embryogenic ability during somatic embryogenesis.

In the pink module (somatic embryo development), 23 hub genes that showed high connectivity weight included 11 TFs representing distinct families, such as the *AP2/ERF-ERF, C2C2, C3H, bHLH, GRF*, and *bZIP* TFs (Supplementary Table 2). Another 12 hub genes included *SMR4, SBP65, LEA6, LIP1*, and others. Based on their BiNGO annotations, some genes associated with SEs development are involved in stress signaling pathways and RNA processes, revealing the relationship between SE development and stress signaling. The rRNA gene are an important part of protein synthesis and ribosome production, and the change of rRNA can cause the change of nucleolus volume (Koukalova et al., 2005). Gaudino and Pikaard (1997) reported that exogenous application of cytokinin stimulated polymerase I transcription of rRNA genes in *Arabidopsis* (Gaudino and Pikaard, 1997). LEA proteins were first identified in cotton seeds more than 30 years ago (Danyluk et al., 1998) and were later found to be abundantly expressed in late zygotic embryogenesis in many plant species, including cotton, barley, rice, oilseed rape, and wheat (Dure et al., 1989). *LEA* gene was highly expressed in carrot callus and somatic embryo stage (Kiyosue et al., 1992). We speculated these genes may regulate the synthesis and accumulation of cellular proteins and small RNA through some stress signals, thus affecting somatic embryo development and promoting somatic embryo maturation.

## Conclusion

According to our main findings of the hybrid sweetgum SE DEGs and their related networks (K-mean, WGCNA), in combination with the relevant literature, we herein propose a possible molecular model for hybrid sweetgum SE, as shown in Figure 2. During totipotency acquisition, the molecular pathways of the cell undergo drastic changes. There are 4 candidate genes that seem to affect the maintain of totipotency, like the homeotic transcription factor *WOX9*, an important regulator of plant stem cell fate in the shoot meristem, which also may regulate the stem cell fate in the callus; the *BBM1* transcription factor involved in the growth regulation of organ primordia; the *R2R3-MYB* transcription factors are involved in cell proliferation and elongation, which may promote callus proliferation. ARF proteins are believed to activate or repress the target genes and in the transient and heterologous assays, individual *ARFs* are classified as activators/repressors of the gene transcription. *ARF5* is a activator, which encodes the MONOPTEROS (MP) protein, to be the most highly expressed member of the *ARF* gene family in the embryogenic culture of Arabidopsis. About the early embryo development stage, there are interesting interactions between *MYB15, AGL15, LTP3, WOX11*, and *ABI3*. *MYB15* was involved in drought and salt tolerance and may enhance expression levels of genes involved in abscisic acid (ABA) biosynthesis and signaling, as well as those encoding stress-protective proteins. Therefore, *MYB15* may promote somatic embryogenesis by regulating the dryness stress in early SE. *AGL15* is known to enhance the embryogenic response in somatic cells if ectopically overexpressed. *AGL15* can stimulates *ABI3* expression and also represses the auxin response and interacts with GA metabolism to promotes the generation of somatic embryos. The *LTP* homologous gene has been implicated as a well-known early marker of somatic embryogenesis induction in different systems, being that it is linked to the protoderm layer formation, which exerts a regulatory role in controlling cell expansion during embryo development in developing somatic. The *WOX11* genes participate in the early stages of the formation of callus and adventitious roots, initiating the division of the procambium cells. With the further development of somatic embryos, the need for energy stimulates the high expression of *SMR4, ECP40* and *LEA6*, which are involved in the stress signaling pathway, RNA process and protein synthesis, resulting in the accumulation of proteins and ribosomes in proper embryo cells and promoting the maturation of somatic embryos. Our study is the first to use an integrated approach to study SE in hybrid sweetgum. The results of our study are an important starting point for future analyses and provide new insights useful for exploring alternative strategies and possibilities.

## Data Availability Statement

The data presented in the study are deposited in the NCBI repository, accession number PRJNA762421.

## Author Contributions

JFZ, LK, and JZ designed the study. SQ and RZ conducted the experiments, analyzed the data, and drafted the manuscript. CH, HL, JY, TZ, YF, and SC critically reviewed and improved the manuscript. All authors have read and approved the final version of the manuscript.

## Conflict of Interest

The authors declare that the research was conducted in the absence of any commercial or financial relationships that could be construed as a potential conflict of interest.

## Publisher’s Note

All claims expressed in this article are solely those of the authors and do not necessarily represent those of their affiliated organizations, or those of the publisher, the editors and the reviewers. Any product that may be evaluated in this article, or claim that may be made by its manufacturer, is not guaranteed or endorsed by the publisher.

## Footnotes

1
https://www.plabipd.de/portal/web/guest/mercator4
