# Supplementary material for: Global Transcriptome and Coexpression Network Analyses Reveal New Insights Into Somatic Embryogenesis in Hybrid Sweetgum (Liquidambar styraciflua × Liquidambar formosana)
Source: Front Plant Sci. 2021 Nov 22;12:751866. doi: 10.3389/fpls.2021.751866 (PMC8645980; doi:10.3389/fpls.2021.751866)
Supplement: Supplementary file 1 [file Table_1.DOCX]

Supplementary Table 1: Reads mapped against CDS were used in the calculation. The average FPKM value of three biological replicates was used to determine the number of genes with specific range of expression shown.

| sample name | 0.5<FPKM<1 | 1<FPKM<5 | 5<FPKM<10 | 10<FPKM<100 | 100<FPKM | Total Expressed gene |
| --- | --- | --- | --- | --- | --- | --- |
| NEC | 1773 | 5508 | 3376 | 9861 | 1336 | 21855 |
| EC | 1769 | 5548 | 3266 | 9985 | 1362 | 21930 |
| PEM1 | 1931 | 6045 | 3391 | 10321 | 1294 | 22981 |
| PEM2 | 1770 | 5660 | 3404 | 10613 | 1196 | 22642 |
| GE | 2088 | 5982 | 3181 | 8715 | 1387 | 21352 |
| HE | 2043 | 5731 | 3205 | 8966 | 1426 | 21371 |
| TE | 2013 | 5805 | 3194 | 8577 | 1379 | 20967 |
| CE | 2056 | 5919 | 3227 | 8563 | 1330 | 21095 |
