# Supplementary material for: Global Transcriptome and Coexpression Network Analyses Reveal New Insights Into Somatic Embryogenesis in Hybrid Sweetgum (Liquidambar styraciflua × Liquidambar formosana)
Source: Front Plant Sci. 2021 Nov 22;12:751866. doi: 10.3389/fpls.2021.751866 (PMC8645980; doi:10.3389/fpls.2021.751866)
Supplement: Supplementary file 3 [file Table_3.DOCX]

Supplementary Table 3: Gene-speciﬁc primers sequences of qRT-PCR

| Gene_id | Perimer | Primer sequence (5' to 3') |
| --- | --- | --- |
| EVM0020840 | *GLP1-13*-q-F | TCCACACTCCCAGAGACACA |
|  | *GLP1-13-*q-R | TATGGGGAGGGTTTAGGCCA |
| EVM0026553 | *ARF5-*q-F | TGGGACCTTTTCATCTGCCC |
|  | *ARF5-*q-R | TTATGGCGTCAGGTTGGTCG |
| EVM0020968 | *ABI3-1*-q-F | CAACAACAAGAACAGCGGGG |
|  | *ABI3-1-*q-R | GAGCCACTCGAAGAACACCA |
| EVM0021586 | *LTP3*-q-F | GCTAATTGCTGTGGAGGGGT |
|  | *LTP3-*q-R | CAGTGGAGGGGCTGATCTTG |
| EVM0013624 | *WOX9*-q-F | CCAATGAGACCCTTGCACTCTA |
|  | *WOX9-*q*-*R | GGCATCACCCACTTGTCCATA |
| EVM0000925 | *LEA6*-q-F | AGAAGTTGAGCGACATGGCA |
|  | *LEA6-*q*-*R | GTGGCTCATGGACATGGTGG |
| EVM0007351 | *SMR4*-q-F | TCGACGGTGATGCAAGAAGG |
|  | *SMR4-*q-R | GGTCCCGCTTCTTCCCAAAT |
| EVM0003818 | *ECP40*-q-F | GAGACCAGGAGCAAGCATCG |
|  | *ECP40-*q-R | TTGAAAACGGACACGACACG |
| EVM0015487 | *MYB36*-q-F | TGGTGGTTTTACTGAGGAGGA |
|  | *MYB36-*q-R | TCAGTTCTCCCTGGCAGTTG |
|  | *EF1-r-07041-*q-F | ACTGCACGGTCATTGATGCT |
|  | *EF1-r-07041-*q-R | AAAGCATGCTCACGGGTCTG |

Supplementary Table 4: Statistics of reads generated by transcriptome sequencing of hybrid sweetgum SE

| sample name | Total Reads | Mapped Reads | Uniq Mapped Reads | Q30 percentage | Alternative Splicing |
| --- | --- | --- | --- | --- | --- |
| NEC1 | 53852216 | 43344772(80.49%) | 41991037(77.97%) | 94.92% | 45308 |
| NEC2 | 54368240 | 43606590(80.21%) | 42308440(77.82%) | 94.90% | 45047 |
| NEC3 | 54285240 | 43835626(80.75%) | 42275547(77.88%) | 94.72% | 43059 |
| EC1 | 73619080 | 58025117(78.82%) | 56366796(76.57%) | 91.83% | 50227 |
| EC2 | 74287358 | 58026238(78.11%) | 56392267(75.91%) | 91.28% | 48334 |
| EC3 | 64852052 | 50759772(78.27%) | 49168773(75.82%) | 91.08% | 48161 |
| PEM11 | 57801868 | 46029986(79.63%) | 44781459(77.47%) | 93.89% | 49954 |
| PEM12 | 50821524 | 40647673(79.98%) | 39533468(77.79%) | 94.21% | 49238 |
| PEM13 | 50081538 | 40056914(79.98%) | 38992945(77.86%) | 94.08% | 49254 |
| PEM21 | 62463570 | 49089407(78.59%) | 47634973(76.26%) | 93.75% | 49468 |
| PEM22 | 54306422 | 43393813(79.91%) | 42188707(77.69%) | 93.89% | 47233 |
| PEM23 | 52889632 | 41975585(79.36%) | 40761046(77.07%) | 93.71% | 47629 |
| GE1 | 63450314 | 48451523(76.36%) | 47112326(74.25%) | 91.11% | 45760 |
| GE2 | 71927550 | 56870197(79.07%) | 55234399(76.79%) | 94.13% | 49621 |
| GE3 | 61745718 | 48511265(78.57%) | 46959108(76.05%) | 93.41% | 48178 |
| HE1 | 56049650 | 43985425(78.48%) | 42721101(76.22%) | 91.75% | 45919 |
| HE2 | 68371066 | 54447243(79.63%) | 52872347(77.33%) | 94.24% | 51015 |
| HE3 | 69991754 | 54629254(78.05%) | 52949798(75.65%) | 91.71% | 47761 |
| TE1 | 45892440 | 36014451(78.48%) | 34955353(76.17%) | 91.63% | 44077 |
| TE2 | 86761922 | 67604387(77.92%) | 65400808(75.38%) | 91.33% | 49229 |
| TE3 | 58636140 | 46316716(78.99%) | 45037645(76.81%) | 92.21% | 46753 |
| CE1 | 57375324 | 45094385(78.60%) | 43727899(76.21%) | 92.09% | 46564 |
| CE2 | 71680026 | 56735770(79.15%) | 54862988(76.54%) | 92.28% | 47923 |
| CE3 | 68147592 | 53600759(78.65%) | 52185412(76.58%) | 91.47% | 49214 |
| L1 | 55665856 | 44909310(80.68%) | 43771752(78.63%) | 94.20% | 40789 |
| L2 | 58697660 | 47433140(80.81%) | 46332677(78.93%) | 94.62% | 37151 |
| L3 | 52903502 | 42618609(80.56%) | 41538885(78.52%) | 94.13% | 40715 |
| R1 | 46863540 | 38189210(81.49%) | 37112232(79.19%) | 94.45% | 41902 |
| R2 | 58998932 | 47956038(81.28%) | 46630611(79.04%) | 94.16% | 42967 |
| R3 | 50354836 | 40800220(81.03%) | 39674853(78.79%) | 94.42% | 43031 |
| S1 | 65570024 | 52716621(80.40%) | 51387326(78.37%) | 94.01% | 46994 |
| S2 | 62237316 | 49987332(80.32%) | 48683945(78.22%) | 94.32% | 44216 |
| S3 | 62053356 | 49635612(79.99%) | 48367389(77.94%) | 93.46% | 46243 |
