# Supplementary material for: Global Transcriptome and Coexpression Network Analyses Reveal New Insights Into Somatic Embryogenesis in Hybrid Sweetgum (Liquidambar styraciflua × Liquidambar formosana)
Source: Front Plant Sci. 2021 Nov 22;12:751866. doi: 10.3389/fpls.2021.751866 (PMC8645980; doi:10.3389/fpls.2021.751866)
Supplement: Supplementary file 6 [file Data_Sheet_1.pdf]

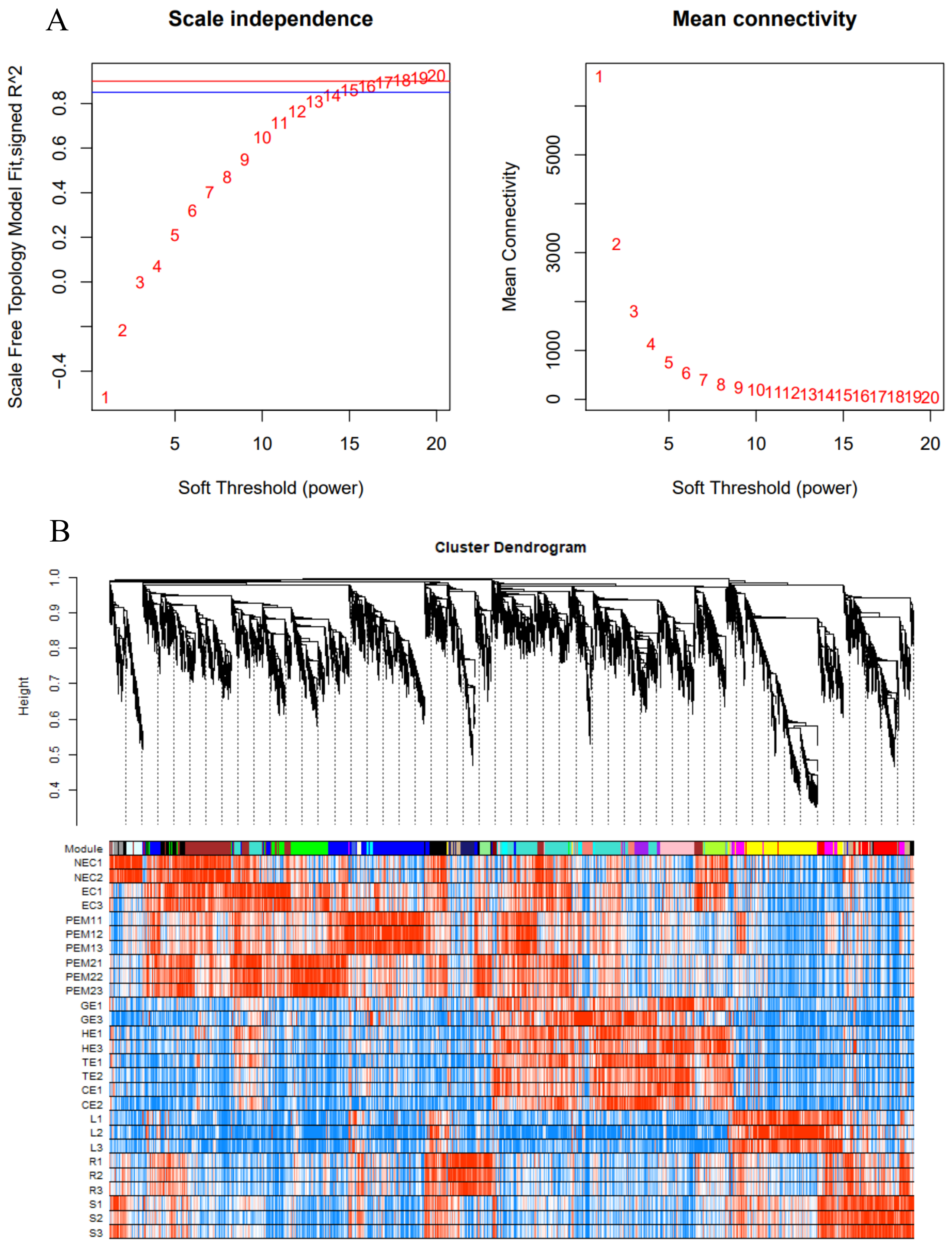

Supplementary Figure 1: Weighted gene co-expression network analysis (WGCNA): **(A)** Scatter determining the soft threshold. **(B)** Hierarchical cluster tree showing 22 modules of co-expressed genes. Each of DEGs is represented by a leaf in the tree, and each of the 22 modules by a major tree branch. The lower panel shows modules in designated colours. The colour shows module-trait correlation from -1 (green) to 1 (red). A high degree of correlation between module and the tissue type is indicated by red. Note that module 'Grey' is for unassigned genes.
