# Supplementary figures and images for: Global Transcriptome and Coexpression Network Analyses Reveal New Insights Into Somatic Embryogenesis in Hybrid Sweetgum (Liquidambar styraciflua × Liquidambar formosana)
Source: Front Plant Sci. 2021 Nov 22;12:751866. doi: 10.3389/fpls.2021.751866 (PMC8645980; doi:10.3389/fpls.2021.751866)

NEC-VS-EC

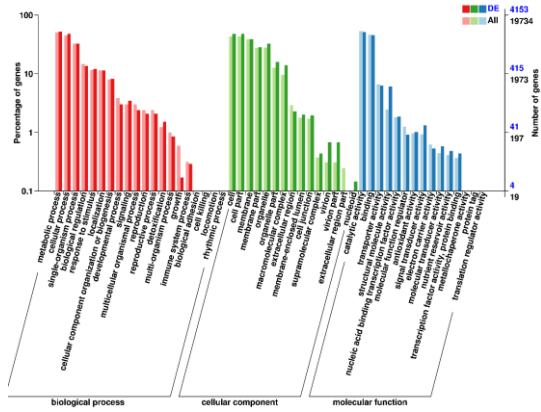

EC-VS-PEM1

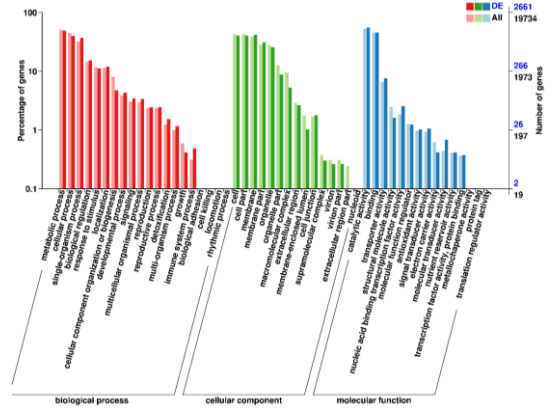

PEM1-VS-PEM2

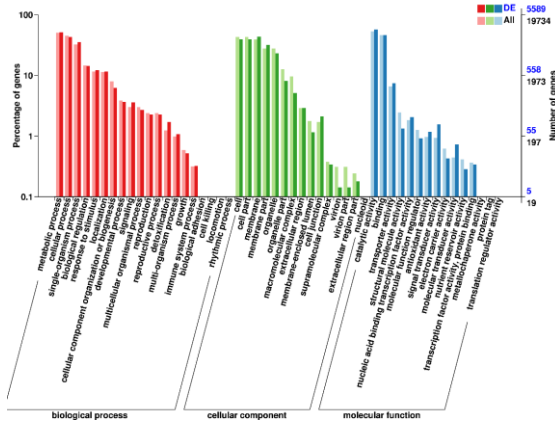

PEM2-VS-GE

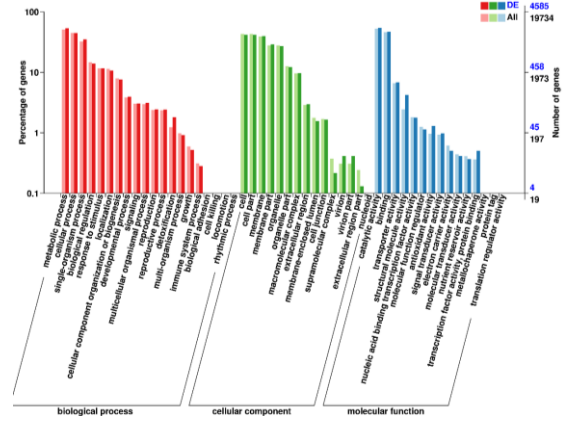

GE-VS-HE

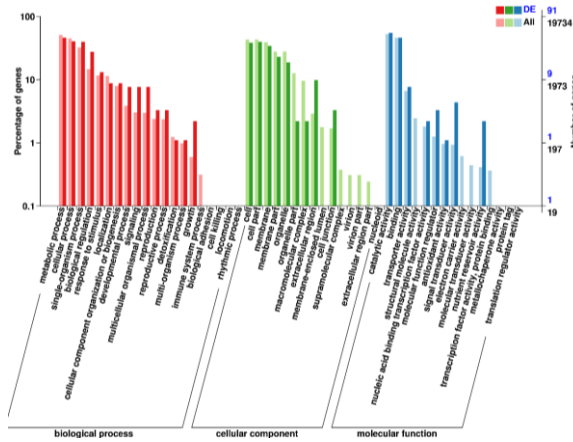

HE-VS-TE

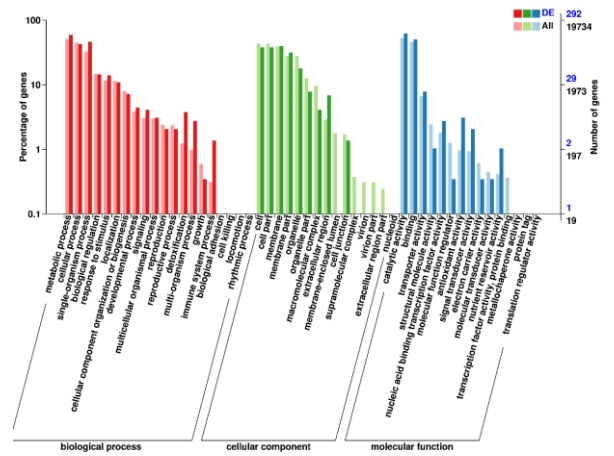

Supplement: Supplementary file 7 [file Data_Sheet_2.PDF]
