# Supplementary material for: Global Transcriptome and Coexpression Network Analyses Reveal New Insights Into Somatic Embryogenesis in Hybrid Sweetgum (Liquidambar styraciflua × Liquidambar formosana)
Source: Front Plant Sci. 2021 Nov 22;12:751866. doi: 10.3389/fpls.2021.751866 (PMC8645980; doi:10.3389/fpls.2021.751866)
Supplement: Supplementary file 8 [file Data_Sheet_3.PDF]

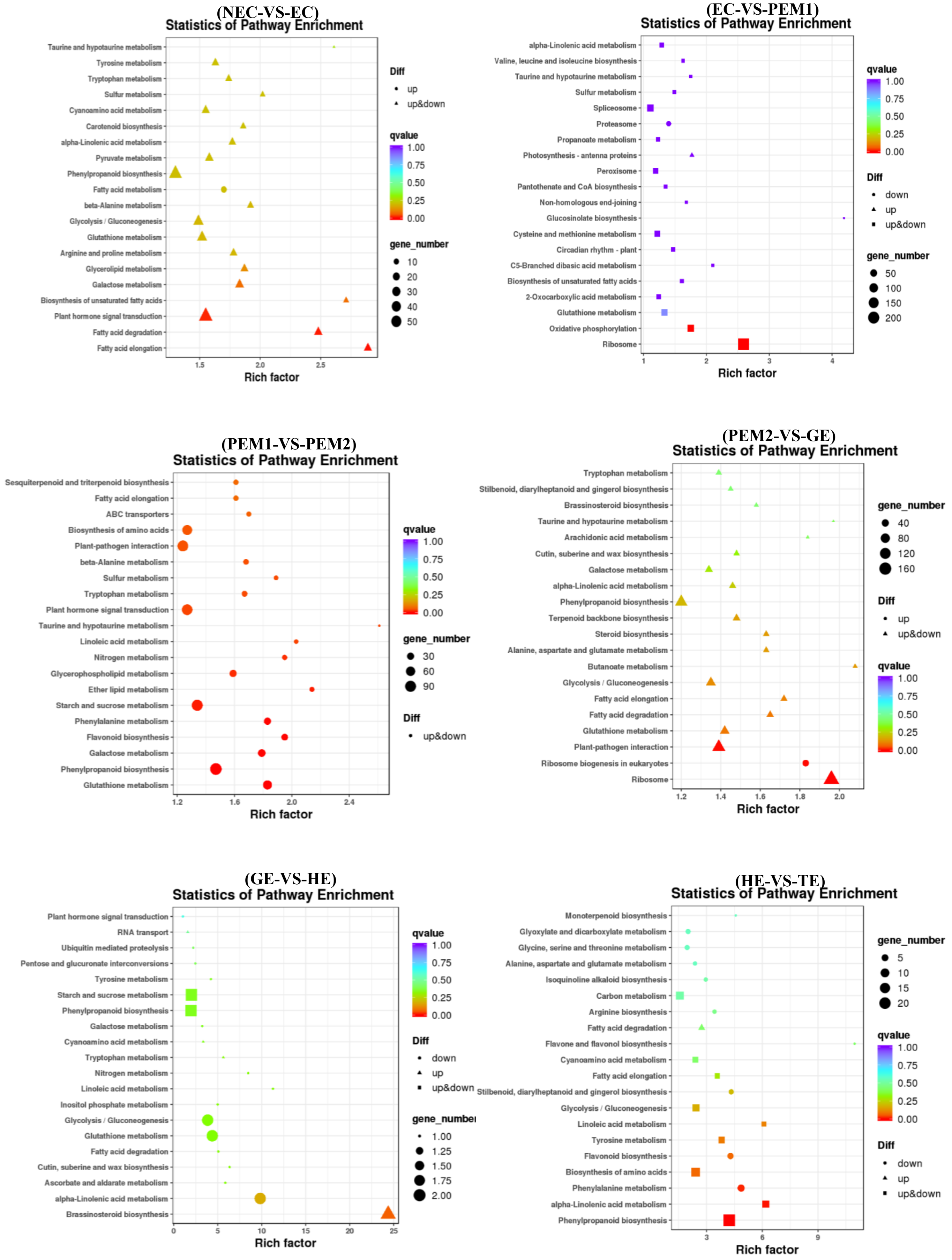
(NEC-VS-EC)
Statistics of Pathway Enrichment
Taurine and hypotaurine metabolism
Tyrosine metabolism
Tryptophan metabolism
Sulfur metabolism
Cyanoamino acid metabolism
Carotenoid biosynthesis
alpha-Linolenic acid metabolism
Pyruvate metabolism
Phenylpropanoid biosynthesis
Fatty acid metabolism
beta-Alanine metabolism
Glycolysis / Gluconeogenesis
Glutathione metabolism
Arginine and proline metabolism
Glycerolipid metabolism
Galactose metabolism
Biosynthesis of unsaturated fatty acids
Plant hormone signal transduction
Fatty acid degradation
Fatty acid elongation
Rich factor
Diff
up
up&down
qvalue
gene_number
(EC-VS-PEM1)
Statistics of Pathway Enrichment
alpha-Linolenic acid metabolism
Valine, leucine and isoleucine biosynthesis
Taurine and hypotaurine metabolism
Sulfur metabolism
Spliceosome
Proteasome
Propanoate metabolism
Photosynthesis - antenna proteins
Peroxisome
Pantothenate and CoA biosynthesis
Non-homologous end-joining
Glucosinolate biosynthesis
Cysteine and methionine metabolism
Circadian rhythm - plant
C5-Branched dibasic acid metabolism
Biosynthesis of unsaturated fatty acids
2-Oxocarboxylic acid metabolism
Glutathione metabolism
Oxidative phosphorylation
Ribosome
Rich factor
qvalue
Diff
down
up
up&down
gene_number
(PEM1-VS-PEM2)
Statistics of Pathway Enrichment
Sesquiterpenoid and triterpenoid biosynthesis
Fatty acid elongation
ABC transporters
Biosynthesis of amino acids
Plant-pathogen interaction
beta-Alanine metabolism
Sulfur metabolism
Tryptophan metabolism
Plant hormone signal transduction
Taurine and hypotaurine metabolism
Linoleic acid metabolism
Nitrogen metabolism
Glycerophospholipid metabolism
Ether lipid metabolism
Starch and sucrose metabolism
Phenylalanine metabolism
Flavonoid biosynthesis
Galactose metabolism
Phenylpropanoid biosynthesis
Glutathione metabolism
Rich factor
qvalue
gene_number
Diff
up&down
(PEM2-VS-GE)
Statistics of Pathway Enrichment
Tryptophan metabolism
Stilbenoid, diarylheptanoid and gingerol biosynthesis
Brassinosteroid biosynthesis
Taurine and hypotaurine metabolism
Arachidonic acid metabolism
Cutin, suberine and wax biosynthesis
Galactose metabolism
alpha-Linolenic acid metabolism
Phenylpropanoid biosynthesis
Terpenoid backbone biosynthesis
Steroid biosynthesis
Alanine, aspartate and glutamate metabolism
Butanoate metabolism
Glycolysis / Gluconeogenesis
Fatty acid elongation
Fatty acid degradation
Glutathione metabolism
Plant-pathogen interaction
Ribosome biogenesis in eukaryotes
Ribosome
Rich factor
gene_number
Diff
up
up&down
qvalue
(GE-VS-HE)
Statistics of Pathway Enrichment
Plant hormone signal transduction
RNA transport
Ubiquitin mediated proteolysis
Pentose and glucuronate interconversions
Tyrosine metabolism
Starch and sucrose metabolism
Phenylpropanoid biosynthesis
Galactose metabolism
Cyanoamino acid metabolism
Tryptophan metabolism
Nitrogen metabolism
Linoleic acid metabolism
Inositol phosphate metabolism
Glycolysis / Gluconeogenesis
Glutathione metabolism
Fatty acid degradation
Cutin, suberine and wax biosynthesis
Ascorbate and aldarate metabolism
alpha-Linolenic acid metabolism
Brassinosteroid biosynthesis
Rich factor
qvalue
Diff
down
up
up&down
gene_numbei
(HE-VS-TE)
Statistics of Pathway Enrichment
Monoterpenoid biosynthesis
Glyoxylate and dicarboxylate metabolism
Glycine, serine and threonine metabolism
Alanine, aspartate and glutamate metabolism
Isoquinoline alkaloid biosynthesis
Carbon metabolism
Arginine biosynthesis
Fatty acid degradation
Flavone and flavonol biosynthesis
Cyanoamino acid metabolism
Fatty acid elongation
Stilbenoid, diarylheptanoid and gingerol biosynthesis
Glycolysis / Gluconeogenesis
Linoleic acid metabolism
Tyrosine metabolism
Flavonoid biosynthesis
Biosynthesis of amino acids
Phenylalanine metabolism
alpha-Linolenic acid metabolism
Phenylpropanoid biosynthesis
Rich factor
gene_number
qvalue
Diff
down
up
up&down

(TE-VS-CE)

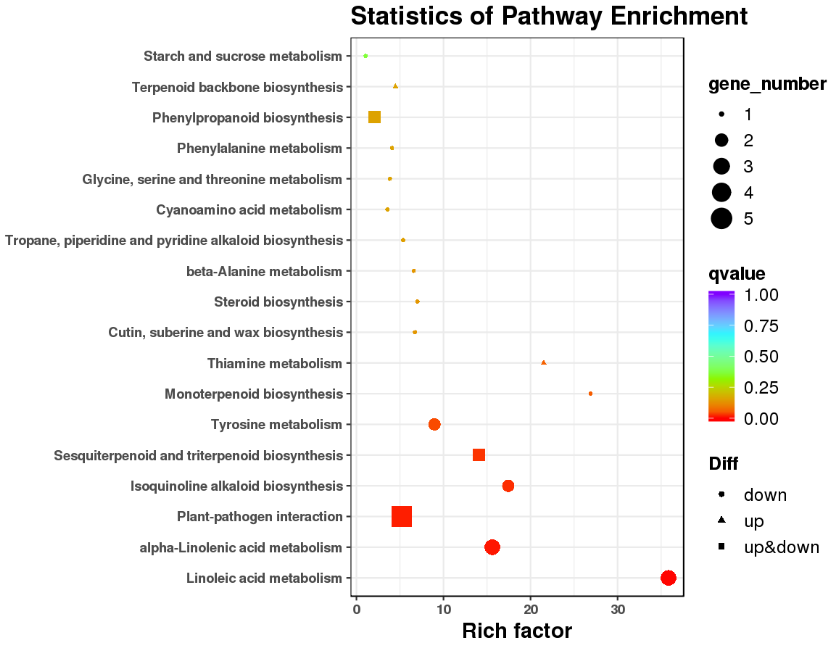

Supplementary Figure 3: Scatterplot of the DEG-enriched KEGG pathway in the pairwise comparisons of NEC_vs_EC, EC_vs_PEM1, PEM1_vs_PEM2, PEM2_vs_GE, GE_vs_HE, HE_vs_TE and TE_vs_CE. Each comparison group takes the first sample period as the control group, and the latter as the experimental group. Rich factor represents the ratio of the number of DEGs and the number of all unigenes in the pathway. The Q value is the corrected P-value.
