# Supplementary material for: Global Transcriptome and Coexpression Network Analyses Reveal New Insights Into Somatic Embryogenesis in Hybrid Sweetgum (Liquidambar styraciflua × Liquidambar formosana)
Source: Front Plant Sci. 2021 Nov 22;12:751866. doi: 10.3389/fpls.2021.751866 (PMC8645980; doi:10.3389/fpls.2021.751866)
Supplement: Supplementary file 9 [file Data_Sheet_4.PDF]

## CRE1

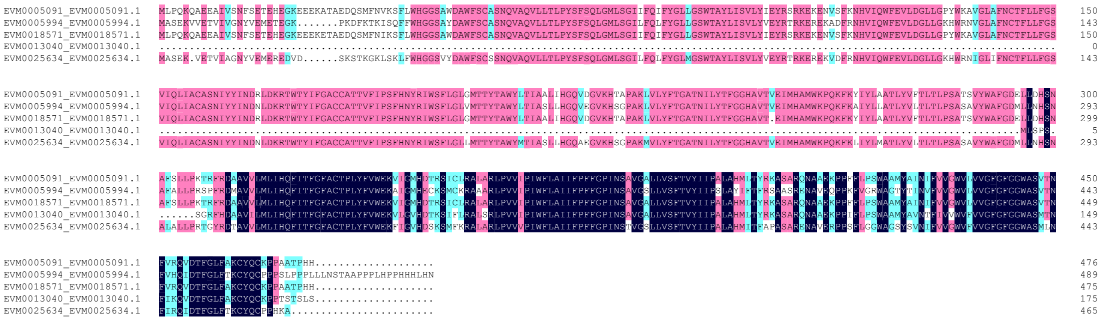

## AUX1（Identity=70.04%）

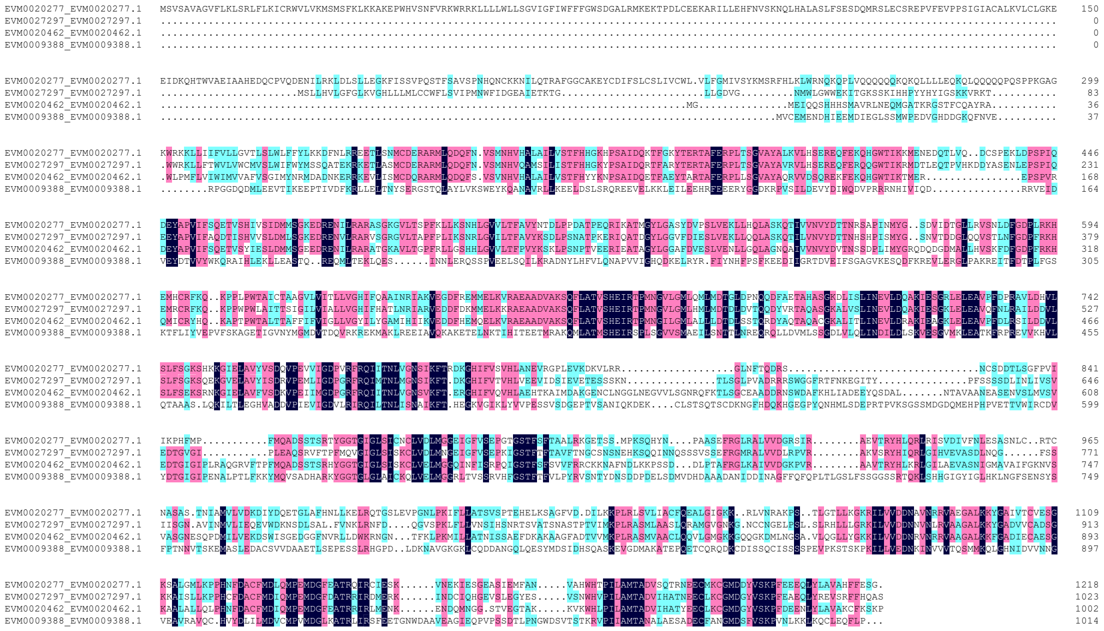

## ABP1（Identity=96.79%）

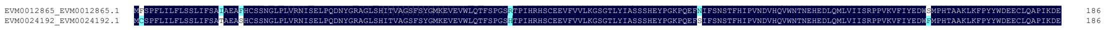

## AFB2（Identity=97.21%）

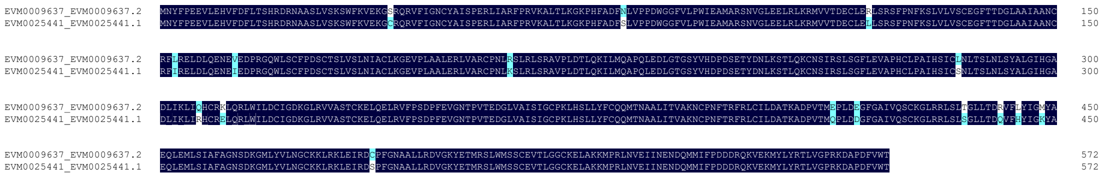

## ASA（Identity=55.08%）

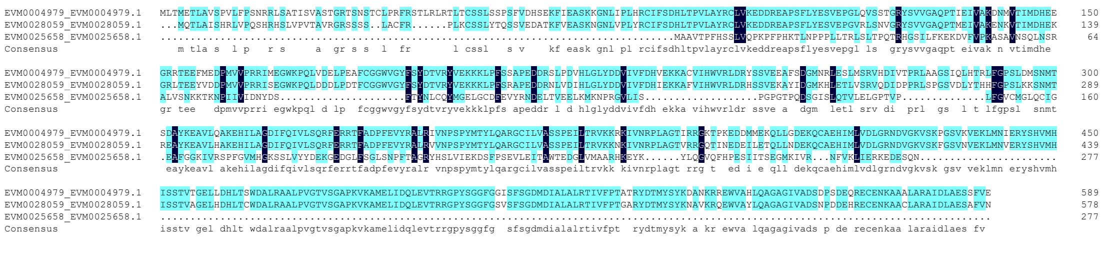

## CKS（Identity=56.06%）

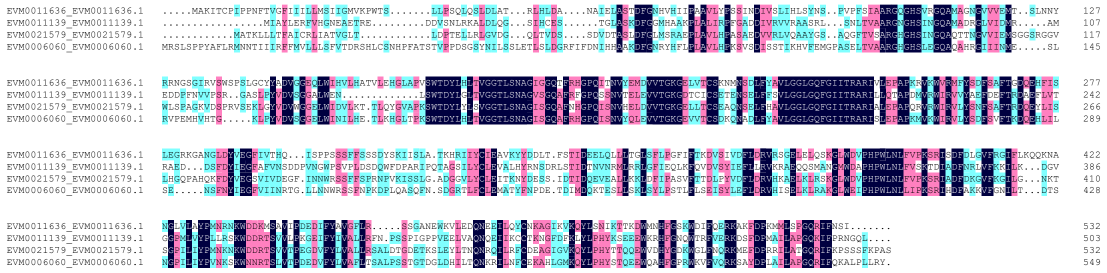

## CYP（Identity=32.48%）

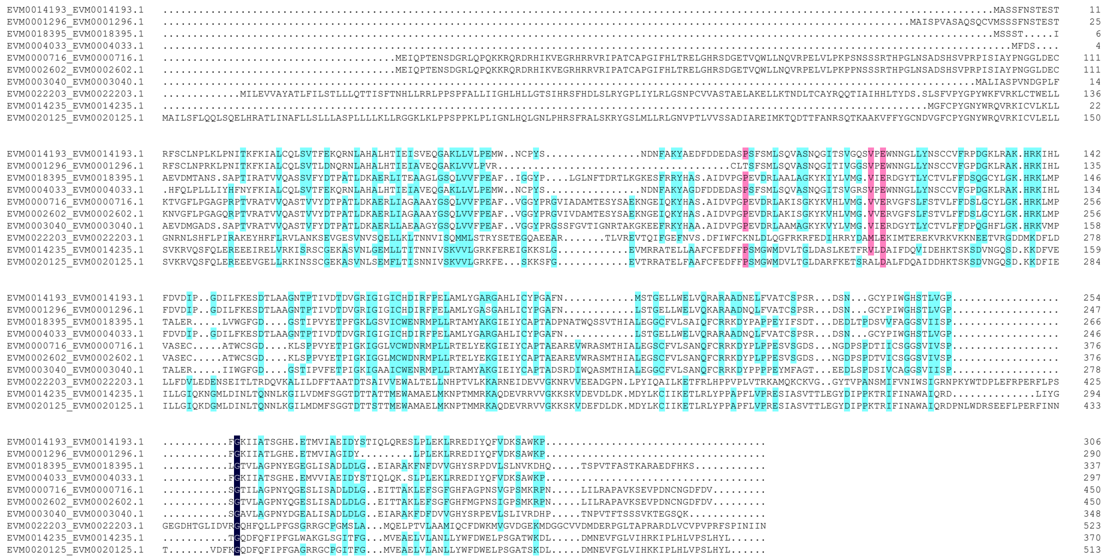

## NIT（Identity=49.47%）

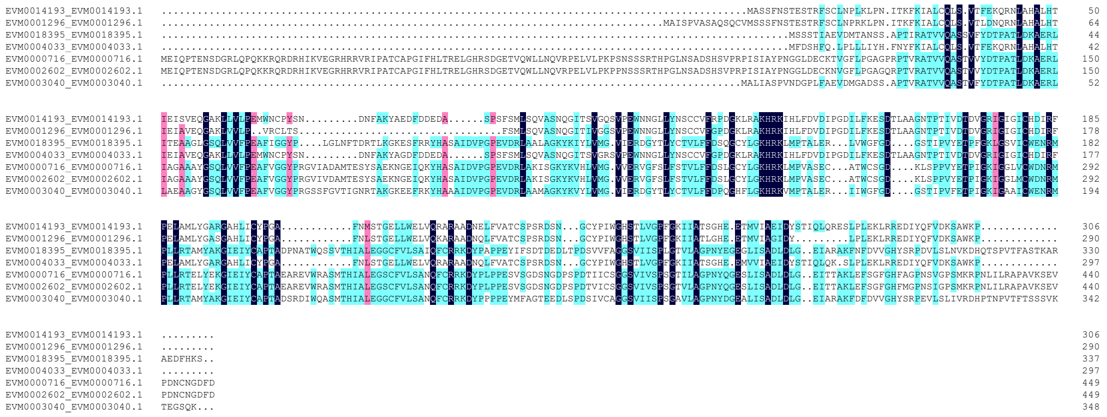

## TPIT（Identity=17.58%）

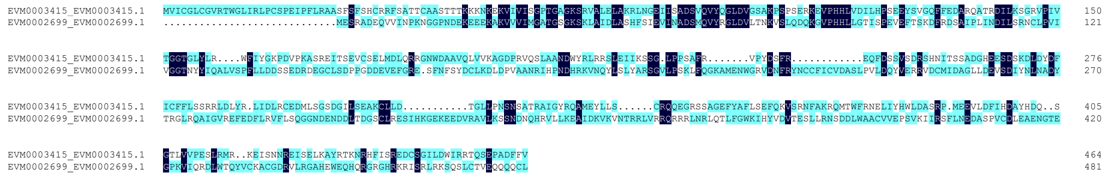

**SUAR（Identity=30.21%）**

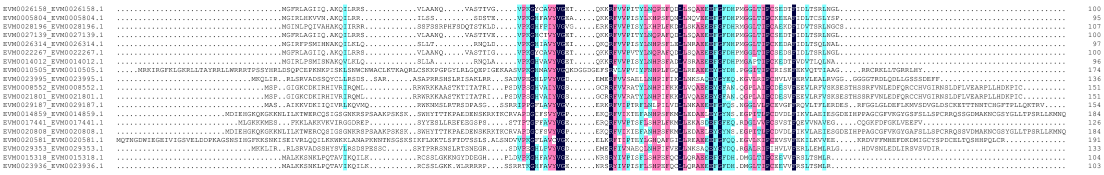

**Supplementary Figure 4:** Multi-sequence alignment of amino acid sequences of similar genes. Black highlights indicate homology levels greater than or equal to 75%, red indicates homology levels greater than or equal to 50%, and blue indicates homology levels greater than or equal to 33%
