# Supplementary material for: Global Transcriptome and Coexpression Network Analyses Reveal New Insights Into Somatic Embryogenesis in Hybrid Sweetgum (Liquidambar styraciflua × Liquidambar formosana)
Source: Front Plant Sci. 2021 Nov 22;12:751866. doi: 10.3389/fpls.2021.751866 (PMC8645980; doi:10.3389/fpls.2021.751866)
Supplement: Supplementary file 10 [file Data_Sheet_5.pdf]

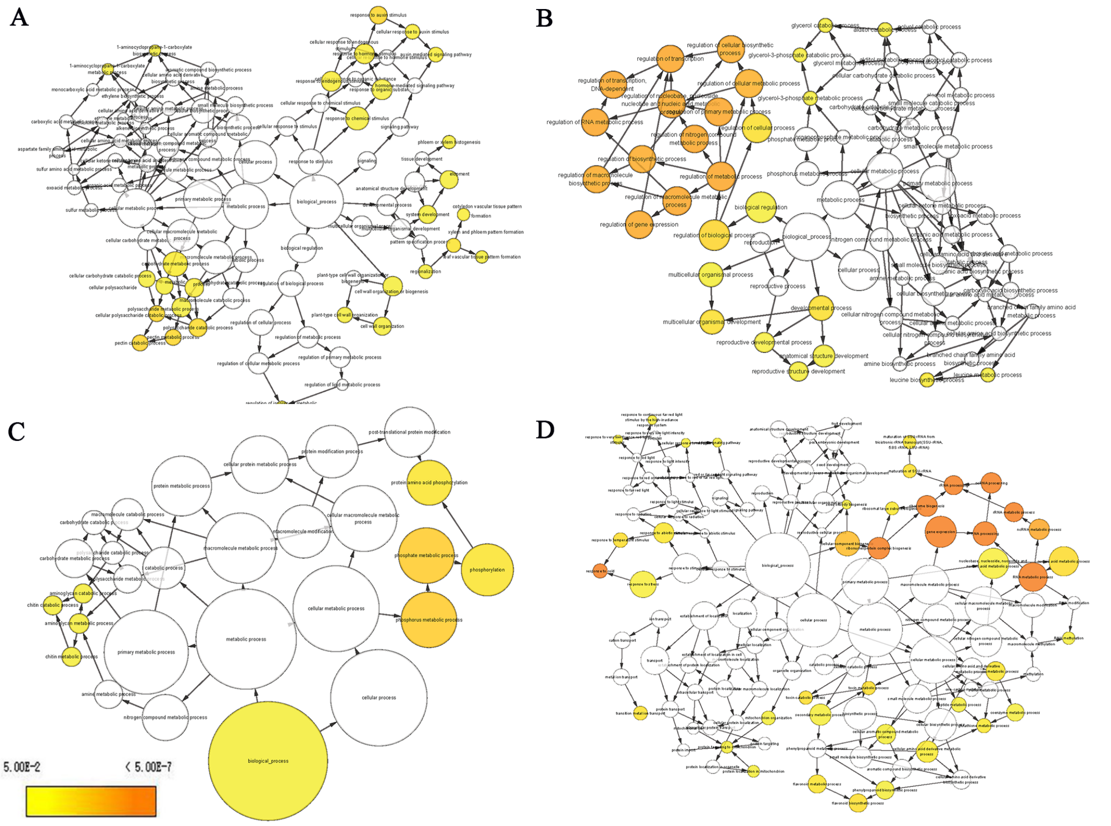

Supplementary Figure 5: Enrichment analysis of DEGs clustered in four module: **(A)** lightcyan module (NEC). **(B)** thistle module (EC). **(C)** blue module (early SE). **(D)** pink (somatic embryo development stages) using GO terms from GO Slim. Significantly over-represented GO terms were visualized by BiNGO application in Cytoscape. The size of a node represents the proportion of the GO term to the number of targets in GO biological process category. The deeper the color, the higher the level of significance.
